# Growth-Stimulatory Effect of Quorum Sensing Signal Molecule *N*-Acyl-Homoserine Lactone-Producing Multi-Trait *Aeromonas* spp. on Wheat Genotypes Under Salt Stress

**DOI:** 10.3389/fmicb.2020.553621

**Published:** 2020-09-29

**Authors:** Muhammad Shoib Nawaz, Ayesha Arshad, Lubna Rajput, Kaneez Fatima, Sami Ullah, Muhammad Ahmad, Asma Imran

**Affiliations:** ^1^National Institute for Biotechnology and Genetic Engineering (NIBGE), Faisalabad, Pakistan; ^2^Plant Physiology and Biotechnology Institute, Agriculture Research Centre, Tandojam, Pakistan; ^3^Department of Life Sciences, University of Management and Technology, Lahore, Pakistan; ^4^Department of Botany, Women University of Azad Jammu & Kashmir, Bagh, Bagh, Pakistan

**Keywords:** AHLs, wheat, *Aeromonas*, PGPR – plant growth-promoting rhizobacteria, halophilic

## Abstract

Salinity is one of the major threats to agricultural productivity worldwide. Soil and plant management practices, along with inoculation with plant-beneficial bacteria, play a key role in the plant’s tolerance toward salinity stress. The present study demonstrates the potential of acyl homoserine lactone (AHL)-producing plant growth promoting rhizobacteria (PGPR) strains of *Aeromonas* sp., namely, SAL-17 (accession no. HG763857) and SAL-21 (accession no. HG763858), for growth promotion of two wheat genotypes inherently different for salt tolerance potential. AHLs are the bacterial signal molecules that regulate the expression of various genes in bacteria and plants. Both *Aeromonas* spp., along with innate plant-growth-promoting (PGP) and salt tolerance traits, showed AHL production which was identified on tandem mass spectrometry as C6-HSL, 3-OH-C5-HSL, 3-OH-C6-HSL, 3-oxo-C7-HSL C10-HSL, 3-oxo-C10-HSL, 3-OH-C10-HSL, 3-oxo-C12-HSL and C6-HSL, and 3-oxo-C10-HSL. The exogenous application of purified AHLs (mix) significantly improved various root parameters at 200 mM NaCl in both salt-sensitive (SSG) and salt-tolerant (STG) genotypes, where the highest increase (≈80%) was observed where a mixture of both strains of AHLs was used. Confocal microscopic observations and root overlay assay revealed a strong root colonization potential of the two strains under salt stress. The inoculation response of both STG and SSG genotypes was evaluated with two AHL-producing strains (SAL-17 and SAL-21) and compared to non-AHL-producing *Aeromonas* sp. SAL-12 (accession no. HG763856) in saline (EC = 7.63 ms/cm^2^) and non-saline soil. The data reveal that plants inoculated with the bacterial consortium (SAL-21 + SAL-17) showed a maximum increase in leaf proline content, nitrate reductase activity, chlorophyll a/b, stomatal conductance, transpiration rate, root length, shoot length, and grain weight over non-inoculated plants grown in saline soil. Both STG and SSG showed relative effectiveness toward inoculation (percent increase for STG: 165–16%; SSG: 283–14%) and showed a positive correlation of grain yield with proline and nitrate reductase activity. Furthermore, principal component analysis (PCA) and categorical PCA analysis clearly showed an inoculation response in both genotypes, revealing the effectiveness of AHL-producing *Aeromonas* spp. than the non-AHL-producing strain. The present study documents that the consortium of salt-tolerant AHL-producing *Aeromonas* spp. is equally effective for sustaining the growth of STG as well as SSG wheat genotypes in saline soil, but biosafety should be fully ensured before field release.

## Introduction

Salinity is edaphic stress that has affected 45 million hectares out of 230 million hectares of irrigated land, causing annual losses of about US$ 12 billion worldwide (FAO, 2020), and is a major threat to global agricultural productivity. There are two types of salinity: primary salinity which occurs in arid and semi-arid regions due to low average rainfall, excessive weathering of rocks, and improper drainage in soils containing high salt contents (Bui, 2013) and secondary salinity which is mainly caused by human activities such as land clearing, inappropriate irrigation practices, and excessive use of chemical fertilizers (de Wit et al., 2011). Salinity decreases the agricultural production of all major crops and deteriorates the structure and the ecological functioning of the soil. It imposes ion toxicity, osmotic and oxidative stresses, limits water uptake from soil, consequently causing nutrient deficiency, especially phosphorous (P) because P ions precipitate with Ca ions (Bano and Fatima, 2009). Salinity also affects photosynthetic efficiency, leaf area, stomatal conductance, and chlorophyll contents.

Plants have also co-evolved the adaptation mechanisms (Gupta and Huang, 2014) against salinity. The first phase of plant response to salinity is characterized by the release of phytohormones, mainly abscisic acid (Ismail et al., 2014), the expression of reactive oxygen species (ROS)-scavenging enzymes (Bharti et al., 2013), and the accumulation of osmoprotectants such as proline (Sneha et al., 2013; Iqbal et al., 2014). The exogenous application of nitric oxide (NO) and nitrate reductase-mediated NO production are also reported as abiotic stress coping strategy in plants, such that they are involved in the homeostasis of ROS in plants (Sun et al., 2015; Pan Q.-N. et al., 2019). The second phase of plant response is characterized by Na^+^ exclusion from xylem parenchyma cells *via* plasma membrane porter HKT1 (Lv et al., 2012; Munns et al., 2012), SOS 1 Na^+^/H^+^ antiporter (Ariga et al., 2013), Na^+^ storage into vacuoles *via* vacuolar Na^+^/H^+^ antiporter (Kronzucker and Britto, 2011), or Na^+^ compartmentalization (Garcia de la Garma et al., 2015).

Many strategies to induce salinity tolerance in plants have been discussed, including genetic engineering of regulatory elements, manipulation of ion transport and transporters, membrane transports, RNAi technology, QTLomics, alternative splicing, and exploring the halobiomes as a gene pool for conferring salt tolerance (Wani et al., 2020). Halobiome is referred to as a group of halophilic and/or halotolerant bacteria, algae, fungi, and plants that can withstand a high-saline environment. It is now generally accepted that plant performance and activities can only be characterized and understood completely if the plant, plus the intimately associated microbiota, is considered. The role of microorganisms in plant growth promotion, nutrient management, and disease control is well established (Naqqash et al., 2020). These microorganisms colonize the rhizosphere/endorhizosphere of plants and promote the growth of plants through various direct and indirect mechanisms (Lugtenberg and Kamilova, 2009; Yang et al., 2009; Upadhyay et al., 2012). The term induced systemic tolerance has been proposed for plant growth promoting rhizobacteria (PGPR)-induced physical and chemical changes that result in enhanced tolerance to abiotic stress. Hence, PGPR acts as an effective strategy to mitigate the detrimental effects of stress along with improved plant growth. PGPR inoculation improves nutrient uptake under stress (Dodd and Pérez-Alfocea, 2012; Han et al., 2014), e.g., *Pseudomonas* sp. inoculation enhances chlorophyll content in maize under salinity while *Klebsiella oxytoca* inoculation improves nutrient uptake in cotton (Nadeem et al., 2007; Wu et al., 2014). However, the inoculation efficiency is higher under normal conditions compared to the stressed condition because stress not only affects the growth and the physiology of plants but also rhizosphere functioning (Hashem et al., 2015). The rhizosphere is a hotspot for microbial diversity and activity and is affected by various abiotic and biotic factors, including nutrients, pH, moisture, and pathogens (Egamberdieva et al., 2010). Microbes native to saline and hypersaline habitats have well-developed physiological pathways and survival mechanisms to cope with the harsh conditions (Torres et al., 2019) and have shown positive effects on plants under salt stress (Ahmad et al., 2015; Singh and Jha, 2016a; Rajput et al., 2018). Under stress conditions, the plant hormone ethylene endogenously regulates plant homeostasis, which results in reduced root and shoot growth. In the presence of 1-aminocyclopropane-1-carboxylic acid (ACC) deaminase-producing bacteria, plant ACC is sequestered and degraded by bacterial cells to supply nitrogen and energy. Furthermore, by removing ACC, the bacteria reduce the deleterious effect of ethylene, ameliorating stress and promoting plant growth.

Acyl homoserine lactones (AHLs) are quorum sensing (QS) molecules produced by root-associated bacteria and represent novel elicitors or inducers of biotic and abiotic stress tolerance in plants. They induce rapid changes in the morphology, physiology, and gene expression of roots and shoots (Schikora et al., 2016), trigger a collective response to change cell density (Papenfort and Bassler, 2016), produce antifungal/antimicrobial molecules and antibiotics (Chapalain et al., 2013), influence colonization and association with the host, and induce host defense mechanism against pathogens (González and Marketon, 2003). QS-related studies from saline and hypersaline habitats have been mainly reported from family *Halomonadaceae* (Llamas et al., 2005; Tahrioui et al., 2013) and recently *Desulfovibrio vulgaris* and *Desulfobacterium corrodens* (Sivakumar et al., 2019). None of the studies reported on AHL production from PGPR strains isolated from saline and hypersaline rhizosphere.

*Aeromonas* spp. have been reported as PGPR and/or biocontrol agent from the rhizosphere of different crop plants, including rice (Mehnaz et al., 2001; Sánchez-Matamoros et al., 2018), soybean (Safni and Antastia, 2018), bean and cotton (Inbar and Chet, 1991), chickpea and mustard (Kundu et al., 2009), and wheat (Chibani et al., 2016; Rajput et al., 2018), but AHL-producing PGPR *Aeromonas* sp. have not been studied until now. AHL-producing *Aeromonas hydrophila* KOR1 was isolated from mangrove rhizosphere (Yin et al., 2015), *Aeromonas caviae* strain YL12 was from plant-based compost material (Lim et al., 2014), and *Aeromonas sobria* was from the spoilage of *Scophthalmus maximus* L. (Li et al., 2016), but these strains were not characterized as PGPR.

This study was based upon the hypothesis that AHL-producing plant-beneficial bacteria may serve as inducers of salt tolerance in plants with concomitant plant growth promotion. The present study has demonstrated the production of different AHLs, varying in acyl chain length (C5–C12), from halo-tolerant, plant-beneficial *Aeromonas* spp. strains isolated from wheat rhizosphere and their subsequent growth-promoting effect on two wheat genotypes (salt tolerant and salt sensitive) under salt stress. Plant inoculation further showed their root colonization potential in saline and non-saline soil. Our results provide evidence that AHLs modulate root architecture, and the inoculation of both AHL-producing *Aeromonas* spp. shows an elevated effect than that of non-AHL strain for plant growth and yield under salt stress. Therefore, the utilization of these bacteria as biofertilizer offers a sustainable solution for crop (wheat) cultivation in saline lands.

## Materials and Methods

### Bacterial Strains and Wheat Genotypes Used

Three test strains *Aeromonas* spp., [SAL-12 (accession no. HG763856), SAL-17 (accession no. HG763857), SAL-21 (accession no. HG763858)], biosensor strain *Chromobacterium violaceum* CV026, reference strain *Rhizobium leguminosarum* strain 8401, and *R. leguminosarum* A34 which is a derivative of strain 8401 containing symbiotic plasmid pRL1J1, along-with the wheat genotypes NW-10-1111-7 (salt-tolerant genotype; STG), and NW-5-1212-I (salt-sensitive genotype; SSG) are mentioned in Table 1 with a short description and growth conditions. The *16S rRNA* gene sequences of *Aeromonas* spp. strains SAL-17, SAL-21, and SAL-12 were aligned to highly similar sequences using multiple sequence alignment, and phylogeny was determined by maximum likelihood method (Jukes and Cantor, 1969) using a MEGA6 software package (Kumar et al., 2016).

**TABLE 1:** Bacterial strains, growth conditions, and wheat genotypes used in this study.

**Strain**	**Description**	**Growth conditions**	**Purpose**	**Source/References**
*Chromobacterium violaceum* CV026	mini-Tn5 mutant of ATCC 31532; violacein negative	LB + kanamycin (25 μg/ml), 28 ± 2°C	Biosensor/indicator strain for AHLs detection; detect and respond to AHLs (C_4_–C_8_ in length) by producing purple pigment violacein	McClean et al., 1997
*Rhizobium leguminosarum* A34	Derivative of strain 8401; carries a symbiotic plasmid pRL1J1	YEM/TY, 28 ±2°C	Reference strain for AHLs production; produce C_4_–C_8_HSLs	Downie et al., 1983
*Aeromonas* sp. strain SAL-17	Wheat rhizosphere isolates from Biosaline Research Station-II (BSRS-II) Pakka Anna (31°24/N and 73°05/E)	LB, 28 ± 2°C	Test strains	Rajput et al., 2018
*Aeromonas* sp. strain SAL-21	Wheat rhizosphere isolates from Biosaline Research Station-II (BSRS-II) Pakka Anna (31°24/N and 73°05/E)	LB, 28 ± 2°C	Test strains	Rajput et al., 2018
*Aeromonas* sp. strain SAL-12	Wheat rhizosphere isolates from Biosaline Research Station-II (BSRS-II) Pakka Anna (31°24/N and 73°05/E)	LB, 28 ± 2°C	Negative strain for AHLs	Rajput et al., 2018
**Parentage of wheat genotypes used in this study**				
**Wheat genotype**	**Genotype description**	**Parentage/pedigree**	**Origin**	**References**

NW-10-1111-7	Salt tolerant	NARC-241/Bhittai-1111-7	Pakistan	Saleem et al., 2015
NW-5-1212-I	Salt sensitive	NARC 41/Bhittai-18 Pakistan		Saleem et al., 2015

### Biochemical and Physiological Characterization

The *Aeromonas* sp. strains used were already reported as PGPR (Rajput et al., 2018). For further characterization, they were tested for tolerance range for NaCl (0.5–10%), pH (6–8), and temperature (4–42°C). Biochemical tests were carried out as described previously (Miñana-Galbis et al., 2002): Gram staining, motility, glucose oxidation–fermentation, oxidase and catalase activity, production of a brown diffusible pigment, hydrogen sulfide production from cysteine and thiosulfate, acid production from carbohydrates, hydrolysis of urea, and utilization of substrates as sole carbon and energy sources. Arginine dihydrolase, lysine decarboxylase, and ornithine decarboxylase activity (Moeller’s method) were determined as described by Smibert and Krieg (1994). The hemolytic activity of strains was tested by spot inoculating the cells onto nutrient agar plates containing 5% sheep blood. The plates were incubated at 28 ± 2°C and observed for hemolysis.

### Identification and Analysis for AHLs

#### Detection of AHLs

Initial screening of AHL production was done by the overlay assay (McClean et al., 1997). Briefly, 100 μl of an overnight-grown culture of test strain was spot-inoculated onto a Luria broth (LB) agar plate. The indicator strain *C. violaceum* CV026 (mini Tn5 negative mutant for violacein production) was grown individually in TY medium, mixed with semi-solid LB agar (0.7%) cells, and spread onto the test strain, and the plates were streaked with the reference strains. The bacterial strain SAL-12 was used as AHL negative control. The plates were incubated at 28 ± 2°C for 24 h and observed for the development of purple color. The AHLs produced by test strains/reference strains diffused through the agar and stimulated violacein synthesis (blue/purple pigmentation) in *C. violaceum* CV026 which cannot synthesize its own AHLs.

For the confirmation of the AHL system in *Aeromonas*, a primer pair was designed to amplify a ≈750-bp sequence from the regulatory gene of the LuxR-type transcriptional regulator in *Aeromonas* spp. from the sequences available in the database. Lux gene was amplified using the primer pair P1 = 5′-ATGAAACAAGACCAACTGCT-3′/P4 = 5′-AAGCTTAATGCCACTGCTCACC-3′ using the following conditions: initial 5 min denaturation at 95°C, followed by 30 cycles at 95°C for 60 s, 57°C for 30 s, 72°C for 45 s, and a final extension step of 72°C for 10 min.

#### TLC and ESI-MS/MS Analysis of AHLs

Bacterial strains were individually grown at 28 ± 2°C for 3 days in LB broth with constant shaking at 200 rpm. AHLs were extracted twice from spent supernatant using an equal volume of acidified ethyl acetate (0.1% glacial acetic acid v/v) and confirmed by overlay assay as described earlier (Hanif et al., 2020). Extraction and subsequent reverse phase-thin layer chromatography (RP-TLC) of AHLs were performed as described (Imran et al., 2014; Ali et al., 2016) on glass-backed C18 reverse phase plates (Merck) developed with an overlay of the exponentially grown culture of CV026.

For electrospray ionization (ESI) analysis, the AHL extracts were purified by solid phase extraction (SPE) (Li et al., 2006), and ESI–mass spectra were obtained by infusion with 5% formic acid on a mass spectrometer (LTQ XL Linear Ion Trap Mass Spectrometer from Thermo Scientific, United States) equipped with and ESI probe. All conditions were set as described previously (Ali et al., 2016), and data were acquired in positive and negative total ion full-scan mode (mass scan range: *m*/*z* 50–500). Various AHL peaks produced during full scan were subjected to tandem mass spectrometry (MS/MS) to confirm their chemical structures based on the fingerprints of their daughter ion peaks produced during fragmentation. The structures of AHLs and the fragmentation schemes were generated using Chem Bio Draw Ultra 12.0. The functions of AHLs already reported in the literature were assigned to those detected in the present study.

### Plant Inoculation Assays

#### Formulation of Halo-Tolerant PGPR Inoculum

Due to the difference in the AHL production ability and different PGPR activities of both strains (Rajput et al., 2018), the bacterial strains were inoculated individually; a consortium containing bacterial strains SAL-21 and SAL-17 was formulated as well. Before this, both strains were tested for compatibility by a standard well-cut method (Rajendran et al., 2011). After confirmation of compatibility, the bacteria were grown separately in LB medium overnight up to an optical density (OD) of 0.45; the cells were harvested by centrifugation and mixed (1:1 ratio) in 0.85% saline to get a consortium of halo-tolerant bacteria (PGPR-consortium) for plant inoculation. A non-AHL-producing *Aeromonas* sp. strain SAL-12 was used as negative control in pot experiment.

#### Root Colonization and QS Detection Under Induced Salinity Under Monoxenic Condition

Seeds of salt-tolerant wheat genotype (NW-10-1111-7) were surface sterilized with 2% sodium hypochlorite for 5 min, washed thrice with sterile distilled water, and germinated in the dark in sterile plastic plates containing 1% water agar supplemented with 200 mM NaCl at 25 ± 2°C. After germination, 3-day-old seedlings were inoculated with bacterial strains (SAL-17 and SAL-21) and grown for 10 days at day/night temperature of 25/20°C and light/dark periods of 16/8 h. The roots were transferred to new LB agar plates overlaid with biosensor strain CV026 and incubated at 30 ± 2°C. Another experiment was set up with three replicates for root colonization analysis under a confocal laser scanning microscope (CLSM) using the same conditions. The roots were detached from the seedlings after 10 days aseptically and stained for 4–5 min in 20–30 μl methyl acridine orange dye. The roots were washed with sterile water and observed under a CLSM (Fluo view, FV 1000, Olympus) attached with a digital monitoring system for capturing the fluorescence image. The samples were excited using the argon–ion laser line at 502–525 nm (for acridine orange), and fluorescence of the samples was detected. The fluorescent images were captured using FluoView software (Olympus).

#### Effect of AHL Treatment on Wheat Roots Under Axenic Condition

Seeds of both wheat genotypes were surface sterilized with 2% sodium hypochlorite for 5 min and washed thrice with sterile distilled water. Purified AHL mixes (200 μl) from both strains were mixed individually in 15 ml of 0.8% water agar medium and poured as a thin layer onto the water agar plate. For the mix-AHLs treatment, AHL extracts from both strains were mixed in a 1:1 ratio and mixed in water agar before pouring into the plates. Sterilized seeds were placed on the plate and germinated in the dark. The experiment was conducted in a completely randomized design with four replicates each. At 7 days after germination, the seedlings were removed from agar, and the roots were washed with distilled water and scanned using Rhizoscanner (EPSON Perfection V700Photo, Epson America, Inc. United States), equipped with WinRHIZO software (Regent Instruments Co. Canada). The roots were also observed under a light microscope (Leica DMLS) for the development of root hairs, and photographic images were recorded using digital camera.

### Pot Experiment: Effect of AHL-Producing *Aeromonas* spp. on Wheat Growth in Saline Soil

A pot experiment was carried out in sterilized saline soil (BSRS-II) in the wheat growing season. The seeds of wheat genotypes were inoculated separately with AHL-producing *Aeromonas* spp. SAL-17 and SAL-21, a mix of both SAL-17 + SAL-21 (consortium), and non-AHL-producing *Aeromonas* sp. SAL-12. Non-inoculated seeds in saline soil and non-saline soil were set as controls. The experiment was set up in a completely randomized design with five replicates of each treatment, and the plants were grown in natural wheat growing season. The plants were evaluated for different stress-related and agronomic parameters at 45–50 days after germination, while yield data were recorded at maturity.

#### Total Proline Contents

Free proline contents from wheat leaves were measured according to the method of Bates et al. (1973). Fresh leaves (0.5 g) were extracted in 10 ml of 3% sulfosalicylic acid. Then, 2.0 ml of the filtrate was mixed with 2.0 ml of acid ninhydrin, followed by 2.0 ml of glacial acetic acid. The samples were incubated at 100°C for 60 min and cooled in an ice bath, and 4.0 ml of toluene was added to the solution and mixed vigorously. The chromophore-containing toluene was aspirated, and the absorbance read as 520 nm on a spectrophotometer (IRMECO U2020). Proline concentration in the samples was determined from a standard curve and calculated on a fresh weight basis.

#### Nitrate Reductase Activity

Nitrate reductase activity from wheat leaves was measured by homogenizing leaves in a chilled mortar and pestle with 100 mM potassium phosphate buffer (pH 7.4), containing 7.5 mM cysteine, 1 mM ethylenediamine tetraacetic acid (EDTA), and 1.5% (w/v) casein. The homogenate was centrifuged at 10,000 × *g* for 15 min at 4°C. Nitrate reductase activity was determined as described (Robin, 1979). The extract was incubated in a reaction mixture containing 100 mM potassium phosphate buffer (pH 7.4), 10 mM EDTA, 0.15 mM NADH, and 0.1 M KNO_3_ at 30°C for 30 min. The reaction was stopped by 100 mL of 1.0 M zinc acetate. The absorbance of the supernatant was determined at 540 nm after diazotation of nitrite ions with 5.8 mM sulfanilamide and 0.8 mM *N*-(1-naphthyl)-ethylenediamine-dihydrochloride.

#### Chlorophyll Contents and Gas Exchange Parameters

Chlorophyll a and b were determined using 500 mg fresh leaf extracted overnight with 80% acetone and centrifuged at 10,000 × *g* for 5 min. The absorbance of the supernatant was estimated using a spectrophotometer at 480-, 645-, and 663-nm wavelength against the solvent, and chlorophyll contents were calculated according to Arnon (1949).

Measurements of transpiration rate (E) and stomatal conductance (gs) were made on the third leaf from the top of each plant using an infrared gas analyzer (Analytical Development Company, Hoddeson, United Kingdom) on a sunny day from 10 to 11 a.m.

#### Morphological and Field Data

The parameters studied for morphological data at 25 dpi were plant fresh weight and shoot and root length and at 75 dpi were shoot and root (length, fresh weight, and dry weight) and plant biomass along with the weight of 1,000 grains. Five plants from each replicate and 15 plants per treatment were uprooted at maturity, and the mean was calculated for each treatment.

### Statistical Analysis

Data were analyzed statistically by analysis of variance technique, using the Statistix (version 8.1) software, and the least significant difference test (Fisher LSD) at 5% probability was used to compare the differences among treatment means. The data presented in this work are the average of at least 15 plants per treatment; means ± standard deviations are given in the figures. Graphs were constructed using Microsoft Excel (2016) and assembled using Corel Draw (R 12). Pearson/Spearman’s correlations were calculated at 1,000 bootstrap analysis at 0.05 level (two-tailed). Categorical principal component analysis was performed using IBM SPSS software package version 20 (SPSS, Inc. Chicago, IL, United States).

## Results

### Biochemical and Physiological Profiling of *Aeromonas* Species

Cells of *Aeromonas* spp. SAL-21, SAL-17, and SAL-12 are motile and Gram-negative. Growth occurs at 25–37°C, 0–10% NaCl (w/v), and pH 6.5–9.5. Optimum growth temperature is 28 ± 2°C. All three strains are positive for oxidase and catalase tests. The brown pigment is not produced by any species. SAL17 and SAL-12 are positive for alanopine dehydrogenase (ADH) and β-galactosidase tests but negative for lactate dehydrogenase (LDH) and octopine dehydrogenase (ODH). SAL-21 is negative for ADH, LDH, and ODH but positive for the β-galactosidase test. Only SAL-21 cannot hydrolyze urea. All strains produce H_2_S and utilize sodium citrate and malonate except SAL-12. Acid is produced from arabinose, mannitol, sucrose, sorbitol, maltose, succinate, rhamanose, inositol, and melibiose from all *Aeromonas* spp. in this study. The β-hemolytic activity was not found in any strain. All the biochemical and the physiological test results of *Aeromonas* spp. strains have been summarized and compared with the already reported *Aeromonas* species in Table 2 for the phenotypic and the biochemical differentiations. The plant-growth-promoting traits of these *Aeromonas* spp. strains are already published (Rajput et al., 2018), and their phylogenetic tree is shown in Supplementary Figure S1.

**TABLE 2:** Key biochemical and physiological tests for the phenotypic differentiation of *Aeromonas spp.* strains SAL-17 and SAL-21 from reported species of genus *Aeromonas.*

**Characteristics**		**1. *Aerom- onas* sp. SA*L*-17**	**2. *Aerom- onas* sp. SAL-21**	**3. *Aerom- onas* sp. SAL-12**	**4. *A. pisci- cola***	**5. *A. salmoni-cida***	**6. *A. besti- arum***	**7. *A. mollus- corum***	**8. *A. sobria***	**9. *A. bival- vium***	**10. *A. veronii***	**11. *A. jandaei***	**12. *A. hydro- phila***	**13. *A. popo- ffii***	**14. *A. enche- leia***
Cell shape		Rod	Rod	Rod	Rod	Rod	Rod	Rod	Rod	Rod	Rod	Rod	Rod	Rod	Rod
Brown pigment		–	–	–	–	+	–	–	–	–	–	–	–	–	–
Gram’s reaction		–	–	–	–	–	–	–	–	–	–	–	–	–	–
Catalase		+	+	+	+	+	+	+	+	+	+	+	+	+	+
Oxidase		+	+	+	+	+	+	+	+	+	+	+	+	+	+
Motility		+	+	+	+	–	+	+	+	+	+	+	+	+	+
NaCl tolerance (%)		0.5–6.5	0.5–6.5	0.5–6.5	0–3	0–5	0–1	0-3	0–3	0.5–6	0–1	0–3	0–3	0–1	0–3
pH tolerance (%)		6.5–9.5	6.5–9.5	6.5–9.5	6.5–7.5	4–5	6.5–7.5	8.5–9.5	6.5–7.5	5–9	6.5–7.5	8.5–9.5	6.5–7.5	6.5–7.5	8–9
Temperature tolerance (°C)		25–37	25–37	25–37	4–37	4–37	20–37	4–37	30–37	4–37	22–37	4–42	28–37	4–37	4–37
H_2_S Production		–	–	+	+	+	+	–	+	–	–	+	+	±	–
Urea hydrolysis		+	–	+	+	nd	nd	–	–	–	–	nd		–	–
Arginine dihydrolase		–	–	–	+	+	nd	+	–	–	–	+	nd	+	+
Production of acid from	Lactose	+	+	-	–	–	–	–	–	–	–	+	–	–	–
	Arabinose	+	+	+	–	+	+	+	–	+	–	–	+	+	–
	Mannitol	+	+	+	+	+	nd	+	+	+	+	+	+	+	+
	Sucrose	+	+	+	nd	+	+	+	+	+	+	–	+	–	–
	Sorbitol	+	+	+	+	+	–	–	–	–	–	–	–	–	–
	Maltose	+	+	+	+	+	nd	+	nd	+	+	nd	nd	+	+
	Succinate	+	+	+	nd	+	nd		nd	nd	nd	+	nd	+	nd
	Rhamnose	+	+	+	–	–	±	–	–	–	–	–	–	–	–
	Inositol	+	+	+	–	+	nd	–	nd	–	–	–	nd	–	–
	Adonitol	–	–	–	–	+	nd	–	nd	–	–	–	nd	–	nd
	Melibiose	+	+	+	–	+	nd	–	–	–	–	–	–	–	–
	Raffinose	–	–	+	–	+	nd	–	–	–	–	–	–	–	–
Decarboxylation of	Lysine	–	–	–	+	+	+	–	+	+	+	+	+	–	–
	Ornithine	–	–	–	–	–	nd	–	–	–	+	–	–	–	–
Utilization of	Sodium citrate	+	+	nd	nd	–	+	nd	±	+	+	nd	nd	+	nd
	Sodium malonate	+	+	–	nd	–	nd	nd	nd	nd	–	nd	nd	+	nd
Clinical significance		No	No	No	Yes	Yes	Yes	No	No	No	Yes	Yes	Yes	No	No

*Data were taken from 1, 2, and 3 (from this study), 4 (Beaz-Hidalgo et al., 2009), 5 (Austin et al., 1989), 6 (Martínez-Murcia et al., 2005), 7 (Miñana-Galbis et al., 2004), 8 and 12 (Popoff and VéEron, 1976), 9 (Miñana-Galbis et al., 2007), 10 (Hickman-Brenner et al., 1987), 11 (Esteve et al., 2003), 13 (Huys et al., 1997), and 14 (Esteve et al., 1995). +, 80–100% of strains positive; –, 80–100% strains negative; nd, no data available.*

### Analysis of AHLs

**FIGURE 1:**
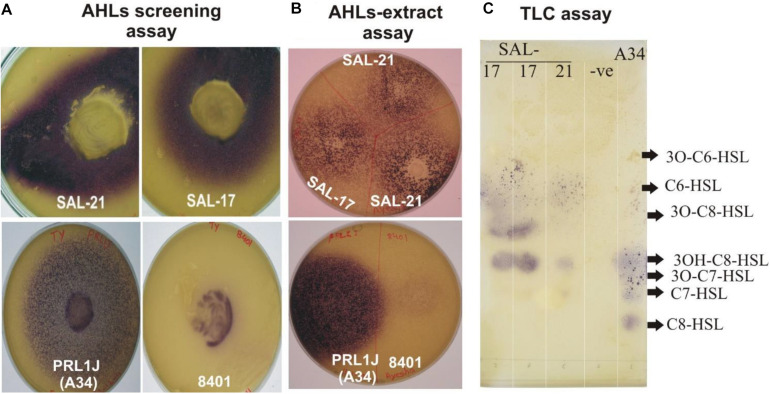
**(A)** Acyl homoserine lactone (AHL) detection assay of *Aeromonas* spp. SAL-21 and SAL-17 with *Chromobacterium violaceum* CV026 showing purple coloration. *Rhizobium leguminosarum* A34 and 8401 refers to positive controls. **(B)** Plate assay of extracted AHLs. **(C)** Reverse phase-thin-layer chromatography of AHL extracts of SAL-17 and SAL-21 with the *C. violaceum* CV026 strain used as a biosensor and the *R. leguminosarum* strain A34 used as a positive control. From right, lane 1—positive control A34, lane 2—solvent control, lane 3—SAL-21, and lanes 4 and 5—SAL-17.

The strains SAL-17 and SAL-21 produced purple color on LB agar plates overlaid with biosensor strain *C. violaceum* CV026, indicating the production of AHLs compared with the positive control (Figure 1A). AHLs were extracted from the cell-free supernatant of strains SAL-17 and SAL-21 and confirmed by plate overlay assay (Figure 1B). The strain SAL-12 did not show any purple color around the colony with the biosensor strain CV026. RP-TLC was further carried out to separate the extracted AHLs (Figure 1C). The comparison was done with the strain *R. leguminosarum* 8401 and a derivative of this strain named A34 containing pRL1J1 as reference for AHLs. Four spots were observed in the lane of SAL-17 and SAL-21 compared to six spots for pRL1J1 (Figure 1C). Both strains (SAL-17 and SAL-21) gave amplification with the *Aeromonas* Lux gene-specific primers, confirming the presence of LuxR-type regulators in them. The AHLs-negative strain SAL-12 did not show any amplification with these primers, indicating the absence of the Lux regulator.

**FIGURE 2:**
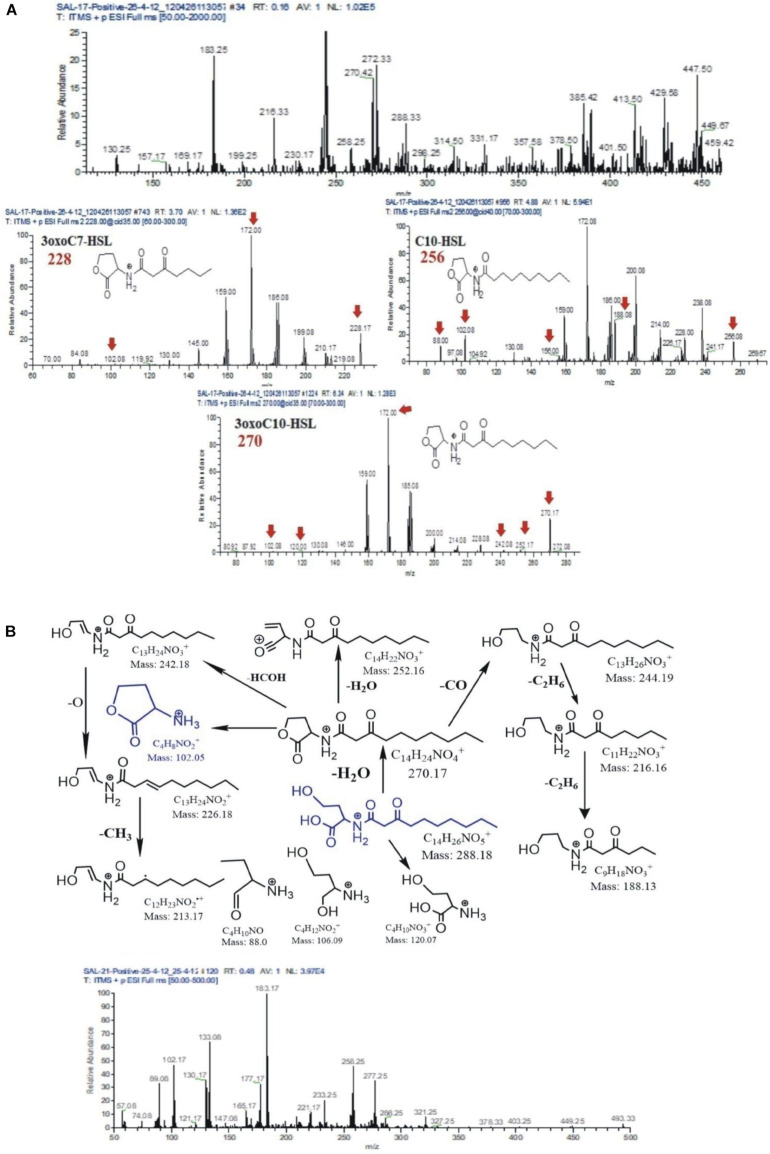
**(A)** Mass spectrometry chromatogram (full MS) with peaks at [M + H]^+^, [M + H + H_2_O]^+^, and [M + Na]^+^ of acyl homoserine lactones (AHLs) extracted from strain SAL17. Protonated AHL [M + H]^+^ peaks were at a very low intensity. The peaks were identified as [M + H + H_2_O]^+^ and [M + Na]^+^ for the presence of AHLs by adding a water molecule or the formation of sodium adducts. **(B)** Fragmentation pattern of AHLs 3oxoC10-HSL m/z 288. All daughter ions generated from the fragmentation of m/z 288 are unambiguously assigned (above). Mass spectrometry chromatogram (full MS) with peaks at [M + H]^+^, [M + H + H_2_O]+, and [M + Na]^+^ of AHLs extracted from strain SAL21 (below). Protonated AHLs [M + H]^+^ peaks were at a very low intensity. The peaks were identified as [M + H + H_2_O]^+^ and [M + Na]^+^ for the presence of AHLs by adding a water molecule or the formation of sodium adducts.

AHL extracts were purified through SPE and were subjected to ESI–mass spectrometry analysis for the profiling of AHLs. The structure of the selected AHLs and their corresponding peaks were confirmed by MS/MS analysis (Tables 3, 4). When an extract of SAL-17 was analyzed, eight AHLs (C6-HSL, 3-OH-C5-HSL, 3-OH-C6-HSL, 3-oxo-C7-HSL, C10-HSL, 3-oxo-C10-HSL, 3-OH-C10-HSL, and 3-oxo-C12-HSL + H_2_O) were observed, and their structures were confirmed by tandem mass spectrometry (Figures 2A,B). Only two AHLs were confirmed by MS/MS analysis, in the case of SAL-21 C6-HSL and 3oxo-C10-HSL + H_2_O (Figures 2A,B); the other two spots detected in TLC could not be detected in MS/MS analysis. Functional annotation of the AHLs was done by equating them with the published literature, and their putative roles were assigned (Table 4).

**TABLE 3:** Liquid chromatography–tandem mass spectrometry analysis of acyl homoserine lactones (AHLs) in spent culture supernatant of *Aeromona*s spp. SAL-17 and SAL-21.

**Sr. #.**	**AHL type**	**m/z (M + H)^+^**	Relative abundance of isolated ions*	**Daughter ions**
1	3-OH-C5-HSL	202	+++	187, 185, 174, 159, 147, 144, 130, 123, 100, 85
2	C6-HSL	200	++	185, 182,172, 158, 156, 144, 130, 114, 102, 88
3	3-OH-C6-HSL	216	+	198, 173, 159, 146, 102, 84
4	3-oxo-C7-HSL	228	++	210, 199, 186, 172, 159, 145, 130, 120, 102, 84
5	C10-HSL	256	++++	238, 228, 214, 188, 186, 172, 159, 130, 102, 88
6	3-oxo-C10-HSL	270	+++	252, 242, 228, 214, 200, 185, 172, 159, 146, 120, 102, 88
7	3-OH-C10-HSL	272	++	254, 228, 214, 200, 186,172, 159, 146, 118, 102
8	3-oxo-C12-HSL	316	+	298, 272, 246, 222, 212, 184, 166, 152, 106, 102
9	C6-HSL	200	+	184, 182, 172, 139, 126, 102, 85
10	3-oxo-C10-HSL	288	++++	273, 270, 260, 244, 214, 188, 174, 160, 144, 140, 125, 106, 102, 88

**TABLE 4:** Functional annotation of acyl homoserine lactones (AHLs) in spent culture supernatant of *Aeromonas* spp. SAL-17 and SAL-21 compared to others reported in literature.

**AHL types detected in this study**	**Putative role/function**	**Detected previously in bacteria**	**Isolated from (host)or tested on plant**	**References**
3-OH-C5-HSL	Putative role in symbiosis	*S. meliloti*	*Alysicarpus bupleurifolius* L. root nodules	Zarkani et al., 2013
C6-HSL	Production/regulation of phenazines, siderophore, chitinases, proteases and pyrrolnitrin, 2,4-DAPG, hydrogen cyanide, antifungal activity against pathogens, induced systemic resistance, systemic induction of ethylene− and salicylic acid-dependent defense-related genes, increased plant resistance to early infection, improved germination, growth, development, and productivity, root elongation, alteration of auxin to cytokinin ratio in roots and shoots, root colonization, synthesis of IAA, elevated defense response	*Pseudomonas* sp., *Burkholderia ambifaria*, *P. chlororaphis*, *Serratia liquefaciens*, *Serratia plymuthica*, *Pseudomonas fluorescens*, *Serratia plymuthica*	Wheat and maize roots, *Lycopersicon esculentum, Brassica napus* L. roots, transgenic *Nicotiana tabacum*, *Arabidopsis thaliana* L. roots, roots of *Brassica napus* subsp. *Napus* L., *Cucumis sativus* L., *Phaseolus vulgaris* L., *Lycopersicum esculentum* L., *Chlorella vulgaris* L. roots	Wood et al., 1997; Chin-A-Woeng et al., 2001; Zhou et al., 2003; Müller, 2006; Schuhegger et al., 2006; Wei and Zhang, 2006; von Rad et al., 2008; Müller et al., 2009; Pang et al., 2009; Bi et al., 2012; Cheng et al., 2018; Hu et al., 2018
	Low concentration protected against salt stress *via* enhanced activity of SOD, POD, CAT, and higher accumulation of MDA, stress-responsive, signal transduction and regulation and biosynthesis-related proteins	NA	*Arabidopsis thaliana* L. Col−0 roots	Ding et al., 2016
	Plant growth promotion including and development of lateral roots and NO accumulation in calyptra, enhanced K^+^ uptake through membrane hyper-polarization	NA	*Hordeum vulgare* L. roots	Rankl et al., 2016
3-OH-C6-HSL, 3-oxo-C7-HSL	Antifungal activity, production of pyrrolnitrin, chitinase, protease siderophores and hydrogen cyanide, rhizosphere colonization, biocontrol activity	*Serratia* sp., *Ochrobactrum* sp.	*Triticum aestivum* L. stems, *Phaseolus vulgaris* L. roots	Liu et al., 2010; Imran et al., 2014
3-oxo-C10-HSL, 3-oxo-C12-HSL	Biofilm formation, expression of defense-related, stress-responsive, flavonoid synthesis, phytohormonal and regulatory genes, salt stress protection mechanism, overall growth promotion	*Pseudomonas putida*, *Sinorhizobium meliloti*, *Pseudomonas aeruginosa*, *Burkholderia graminis*	*Lycopersicon esculentum* L. roots, *Medicago truncatula* L roots, transgenic *Lycopersicum esculentum* L (*Las*I)	Steidle et al., 2001; Steidle et al., 2002; Mathesius et al., 2003; Barriuso et al., 2008
C10-HSL	Post−embryonic root development including lateral and primary root growth and root hair development, adventitious roots formation through H_2_O_2_, NO and cGMP signaling, expression of IAA-responsive genes, induced systemic resistance and root development	NA	*Arabidopsis thaliana* L. roots, *Vigna radiata* L. roots, *Hordeum vulgare* L. roots	Ortíz-Castro et al., 2008; Bai et al., 2012; Hu et al., 2012; Sieper et al., 2014
	Calmodulin-regulated primary root growth	NA	*Arabidopsis thaliana* L. roots	Zhao et al., 2015
	Enhanced activity of critical photosynthetic enzymes including rubisco, maximal and actual photochemical efficiency was also enhanced	NA	*Chlorella vulgaris* roots	Dou et al., 2017
	Increased plant resistance against *B. cinerea via* jasmonic acid signaling under elevated CO_2_	NA	*Lycopersicum esculentum* L. leaves	Hu et al., 2020
3-OH-C10-HSL	Root colonization in microcolonies, plant growth promotion, inhibition of plant defense responses	*Acidovorax radicis* N35	*Hordeum vulgare* L. roots	Han et al., 2016

### Plant Inoculation Assays

#### Root Colonization Under Induced Salinity

**FIGURE 3:**
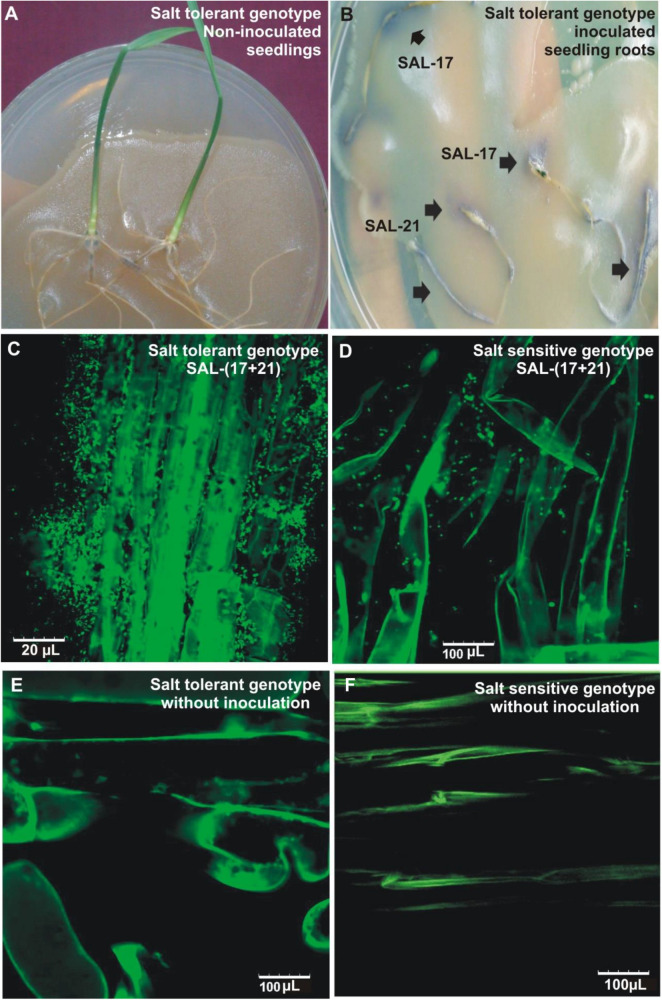
Root colonization of salt-tolerant wheat genotype by bacterial inoculation and subsequent detection by plate overlay assay **(A,B)**. Confocal microscopic images of salt-tolerant **(C)** and salt-sensitive **(D)** wheat roots inoculated with *Aeromonas* spp. bacterial consortium under salt stress. Non-inoculated salt tolerant **(E)** and salt-sensitive **(F)** genotype grown in salt stress are shown as controls.

The development of purple color on the roots shows the bacterial attachment/colonization as seen by the production of AHLs during early seedling growth and root colonization (Figures 3A,B). Root colonization analysis by CLSM, carried out both in the salinized as well as non-salinized medium, showed the colonization of inoculated bacteria on the root surface and root hairs and their presence in close vicinity of the root epidermal cells under salt stress (Figure 3C). A higher number of cells were found on the root surface in the case of wheat grown under salt stress as compared (Figure 3B) to wheat grown under normal conditions (Figure 3D), while non-inoculated control plants did not show the presence of any bacterial cell on root surfaces (Figures 3E,F).

#### Effect of Axenic Supplementation of AHL Extract on Seedling Growth and Root Morphologies

**FIGURE 4:**
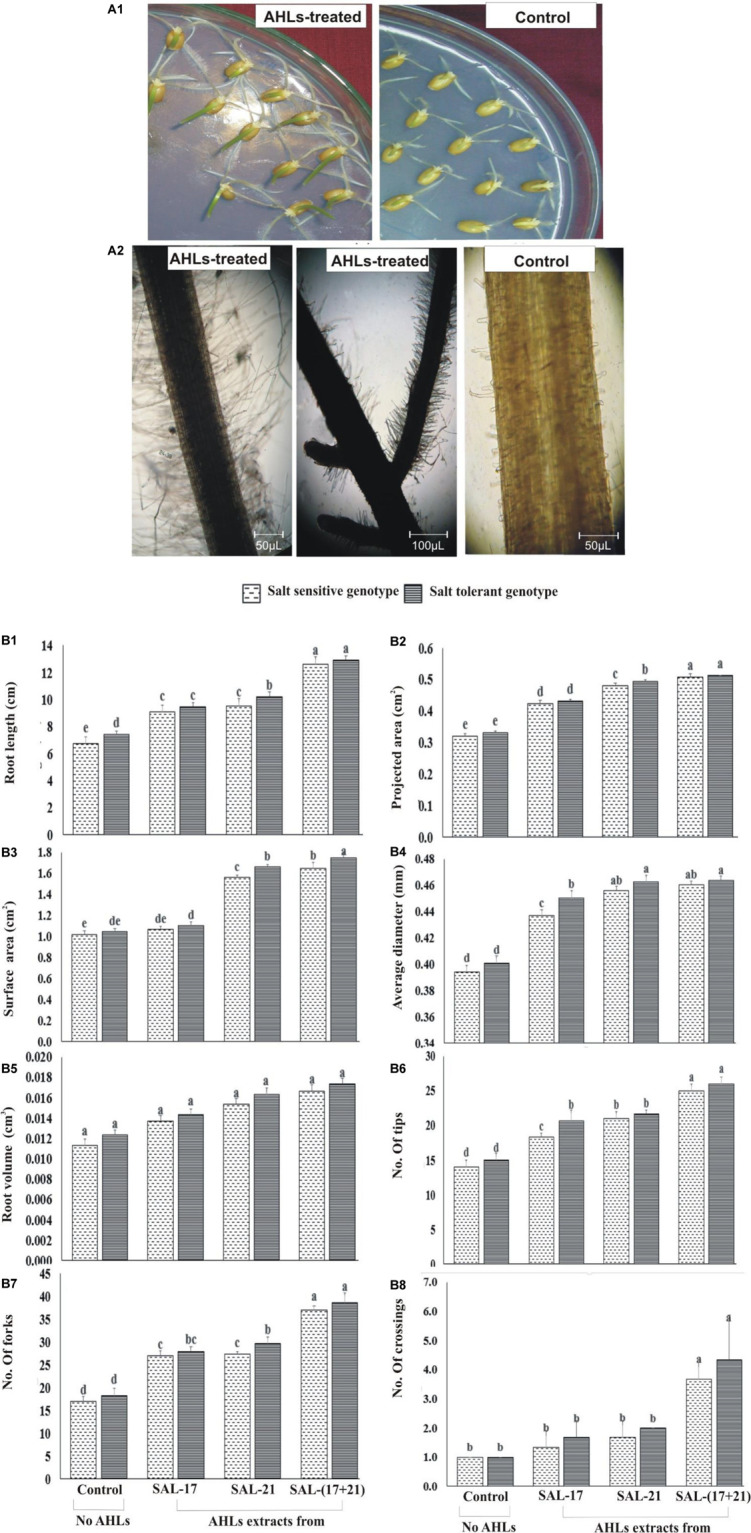
**(A1,A2)** Effect of acyl homoserine lactone (AHL) supplementation on the seedling growth of wheat **(A1)** and AHL-mediated root hair development **(A2)** compared to control wheat seeds grown on water agar. The pictures were photographed at ×100 magnification and then cropped to remove the background. **(B1–B8)** Effect of AHL supplementation on 10-day-old wheat roots under induced salinity (200 mM NaCl) compared to non-treated control in terms of **(B1)** root length, **(B2)** projected area, **(B3)** surface area, **(B4)** average diameter, **(B5)** root volume, **(B6)** number of tips, **(B7)** number of forks, and **(B8)** crossings. Values are the mean of four replicates. Bars represent standard deviation. Values sharing the same letter do not differ significantly (*P* ≤ 0.01) according to Fisher’s least significant difference test.

Wheat seeds grown on AHL-supplemented water agar showed early seedling growth with longer roots and greener shoots compared to the seedlings grown without AHL supplementation (Figure 4A1). Microscopic observations of the root showed the development of more root hairs in roots grown in the presence of AHLs than those grown without AHLs (Figure 4A2).

The rhizoscan data show that the addition of AHLs in agar medium under salt stress significantly improved the root growth and the morphologies in both wheat genotypes (Figure 4B1). Both SSG and STG of wheat, treated either with AHLs of SAL-21 and SAL-17 or a mixture of both, showed a significant increase in different root parameters compared to the non-AHL-treated seedlings. Among all the AHL treatments, seedlings grown on AHL mixture showed the highest percent increase in all root parameters, and AHL extracts of strain SAL-17 showed the lowest. Furthermore, the response of the salt-tolerant genotype was comparatively higher than the salt-tolerant genotype under stress. The AHL-treated salt-sensitive genotype showed an increase of 35–86% in root length (Figure 4B1), 32–58% in projected area (Figure 4B2), 4–61% in surface area (Figure 4B3), 10–16% in average diameter (Figure 4B4), 20–47% in root volume (Figure 4B5), 30–78% in root tips (Figure 4B6), 58–117% in forks (Figure 4B7), and 33–266% in root crossing (Figure 4B8) over non-AHL-treated plants under salt stress, whereas the AHL-treated salt-tolerant genotype showed an increase of 27–74% in root length (Figure 4B1), 30–55% in projected area (Figure 4B2), 5–67% in surface area (Figure 4B3), 12–15% in average diameter (Figure 4B4), 16–40% in root volume (Figure 4B5), 37–73% in root tips (Figure 4B6), 52–110% in forks (Figure 4B7), and 66–333% in root crossing (Figure 4B8) over non-AHLs-treated plants under salt stress.

### Effect of *Aeromonas* spp. Strain Inoculation on Wheat Growth in Saline Soil

#### Proline Contents and Nitrate Reductase Activity

**FIGURE 5:**
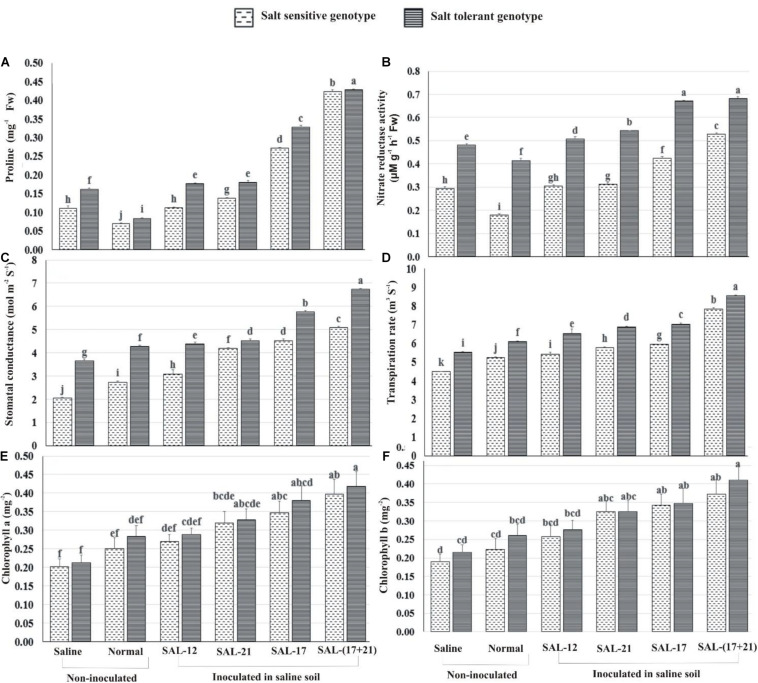
Effect of acyl homoserine lactone-producing and non-producing *Aeromonas* spp. strain inoculation on the biochemical and the physiological parameters of wheat grown in saline soil compared to non-inoculated control: **(A)** proline contents at 45 dpi, **(B)** nitrate reductase activity at 45 dpi, **(C)** stomatal conductance at 45 dpi, **(D)** transpiration rate at 45 dpi, **(E)** chlorophyll a at 50 dpi, and **(F)** chlorophyll b at 50 dpi. Values are the mean of six replicates. Bars represent standard deviation. Values sharing the same letter do not differ significantly [*P* ≤ 0.05 for **(A,B)** and *P* ≤ 0.01 for **(C–E)**] according to Fisher’s least significant difference test.

Proline accumulation and nitrate reductase activity were significantly higher in the leaves of inoculated plants compared to non-inoculated plants grown with and without salt stress in both genotypes. Overall, leaf proline content, nitrate reductase activity, and the photosynthetic performance of inoculated plants in STG were significantly higher than in SSG inoculated plants (Figure 5).

The analysis of treatment response shows that the increase in leaf proline content was maximum in both genotypes where a mixed inoculation of bacteria (consortium) was applied under saline soil, whereas the lowest was observed when both were grown without inoculation under normal soil (Figure 5A). Non-AHL-producing *Aeromonas* sp. SAL-12 also showed an increase in proline contents, but this increase was significantly lower than in the other bacterial inoculation treatments.

Nitrate reductase activity (NR) was significantly higher in STG than in SSG irrespective of bacterial treatments or the soils. The plants inoculated with the bacterial consortium and those inoculated with SAL-17 showed the maximum NR activity and the highest percent increase over the respective non-inoculated control. Similarly, SSG showed maximum NR activity in consortium-inoculated plants, although the activity was much lower than the corresponding treatment in STG (Figure 5B). The leaves inoculated with non-AHL-producing *Aeromonas* sp. SAL-12 showed a little increase in NR activities in both genotypes.

#### Stomatal Conductance, Transpiration Rate, and Chlorophyll Contents

Of the two genotypes, STG exhibited increased activities for all the gas exchange and photosynthetic parameters. The maximum response of inoculation was observed in the treatment where bacterial consortium was applied. In non-inoculated plants, stomatal conductance, transpiration rate, and chlorophyll contents were higher in normal soil compared to those in saline soil (Figures 5C–F).

Stomatal conductance was highest in STG, with an increase of 84% in the consortium, 57.6% in SAL-17, 24% in SAL-21, and 20% in SAL-12 inoculation, respectively, over the non-inoculated control. The salt-sensitive genotype showed an increase of 147.85% in the consortium, 120.36% in SAL-17, 103.57% in SAL-21, and 50.45% in SAL-12 inoculation, respectively (Figure 5C). This shows that, although stomatal conductance was higher in STG, the relative percent increase after inoculation was significantly higher in SSG.

Transpiration rate showed a similar trend and was highest in STG with an increase of 54.5% in the consortium, 27% in SAL-17, 24.5 in SAL-21%, and 18% in SAL-12 inoculation, respectively, over the non-inoculated control. The salt-sensitive genotype showed an increase of 75.05% in the consortium, 32.53% in SAL-17, 29.04% in SAL-21, and 21.26% in SAL-12 inoculation, respectively (Figure 5D).

Chlorophyll a band total chlorophyll contents were significantly high in STG plants after inoculation with the consortium. The improvement in chlorophyll contents was statistically less significant in SSG and other treatments (Figures 5E,F).

In general, the STG showed the maximum increase in all the stress parameters studied, but a comparative analysis of data revealed that the salt-sensitive genotype responded better to inoculation because the percentage increase was higher in SSG than in STG compared to the non-inoculated controls.

#### Growth and Yield

**FIGURE 6:**
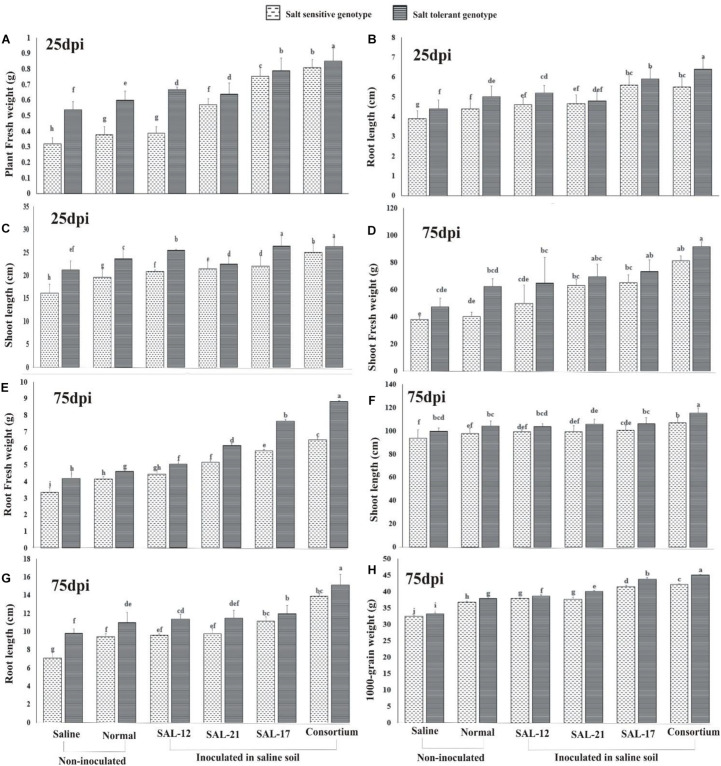
Effect of acyl homoserine lactone-producing and non-producing *Aeromonas* spp. strain inoculation on the growth and the yield of wheat grown in saline soil compared to non-inoculated control: **(A)** plant fresh weight at 25 dpi, **(B)** shoot length at 25 dpi, **(C)** root length at 25 dpi, **(D)** shoot fresh weight at 75 dpi, **(E)** root fresh weight at 75 dpi, **(F)** root length at 75 dpi, **(G)** shoot length at 75 dpi, and **(H)** weight of 1,000 grains (after harvest). Values are the mean of six replicates. Bars represent standard deviation. Values sharing the same letter do not differ significantly (*P* ≤ 0.05) according to Fisher’s least significant difference test.

Analysis of growth and yield parameters of wheat showed a significant increase in inoculated wheat plants compared to non-inoculated controls (Figure 6) in both wheat genotypes. Overall, the salt-tolerant genotype inoculated with bacterial consortium showed the maximum growth and yield, whereas the non-inoculated salt-sensitive genotype in normal or saline soil showed the minimum.

The data regarding plant fresh weight (Figure 6A), root length (Figure 6B), and shoot length (Figure 6C) collected on the 25th day of inoculation showed a significant response of inoculation in both genotypes, although the effect was significantly higher in STG than in SSG. A similar trend was observed for the data collected for growth parameters at 75 days after inoculation for shoot fresh weight (Figure 6D), root fresh weight (Figure 6E), shoot length (Figure 6F), and root length (Figure 6G).

A comparison of treatment means for the 1,000-grain weight (Figure 6H) showed that the response of the salt-tolerant genotype was maximum in all treatments compared with that of the salt-sensitive genotype. Furthermore, the STG plants inoculated with consortium showed the maximum grain weight than all other inoculation treatments.

### The Relationship Among the Parameters

The whole data were subjected for correlation analysis using SPSS, and a direct positive relationship of root length was found with other morphological parameters of root, i.e., projected area (*r* = 0.936^∗∗^), surface area (*r* = 0.876^∗∗^), average diameter (*r* = 0.896^∗∗^), root volume (*r* = 0.912^∗∗^), tips (*r* = 0.903^∗∗^), forks (*r* = 0.958^∗∗^), and crossings (*r* = 0.852^∗∗^). The salt-tolerant genotype showed a specifically higher correlation coefficient ratio (*r* value). Plant fresh weight was found to be positively correlated with other plant morphological (dry weight and length), biochemical (nitrate reductase activity, proline contents), and physiological (chlorophyll contents) parameters (*r* = 0.621^∗∗^–0.958^∗∗^).

Linear regression effectively modeled the positive relationship of grain weight with the chlorophyll contents (*R*^2^ = 0.45 for SSG; *R*^2^ = 0.533 for STG), accounting for 70–82% of the total variance. Quadratic regression was observed for grain weight with nitrate reductase activity (*R*^2^ = 0.858 for SSG; *R*^2^ = 0.880 for STG) and proline contents (*R*^2^ = 0.917 for SSG; *R*^2^ = 0.942 for STG) (Supplementary Figure S2).

**FIGURE 7:**
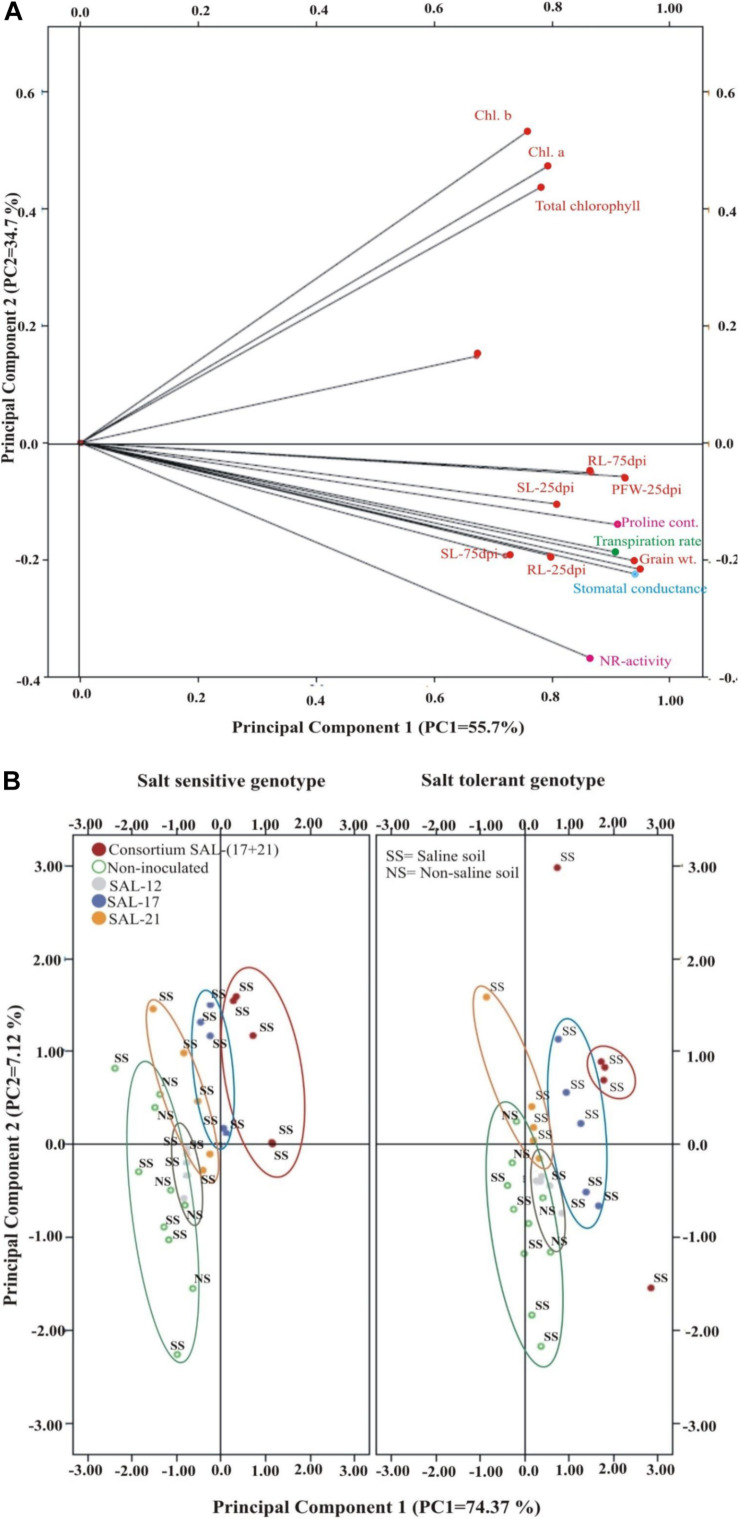
**(A,B)** Categorical principal component analysis (CAT-PCA) of various plant traits measured across salt-tolerant and salt-sensitive genotypes grown in saline soil after different bacterial inoculation treatments (total variance explained: 90%; PC1 = 55.7%, PC2 = 34.7%). CAT-PCA is a non-linear PCA. Factor loadings in PC1 and PC2 are presented as vectors using external scale. PCA showing the response of salt-tolerant and salt-sensitive genotypes toward bacterial inoculation in saline soil compared to non-inoculated controls; loaded as genotypes (total variance explained : 81%; PC1 = 74%, PC2 = 7%).

The CAT-PCA and the PCA captured more than 75–90% of the variance and demonstrated the key genotype difference in both soils and inoculation treatments. The CAT-PCA (Figure 7A) demonstrated that all the observed plant traits/parameters loaded onto the positive quadrant were strongly positively correlated to each other (*R*^2^ = 0.838). The PCA showed that the inoculation response was similar in both genotypes, where the consortium-inoculated plants loaded positively while the non-inoculated plants loaded negatively on PCA (Figure 7B).

## Discussion

Various eco-physiological parameters of soil determine the microbial community and activity in the plant rhizosphere (Egamberdieva et al., 2010). Selection and subsequent plant inoculation of efficient PGPR strains compatible with local eco-physiological conditions can significantly improve a plant’s nutritional status and their overall biotic and abiotic stress tolerance ability. Therefore, we selected wheat rhizosphere isolates from saline soil (BSRS-II) containing multiple plant-growth-promoting traits. They were previously identified as *Aeromonas* spp. with plant-growth-promoting traits (Rajput et al., 2018). The strains were clinically non-significant (negative for the beta-hemolytic reaction) and hence could be used for further studies. Phylogenetic analysis showed their relatedness, but biochemical comparison showed their key differences from other *Aeromonas* spp. strains (Table 1).

This study reports two AHL-producing *Aeromonas* spp. from saline-soil rhizosphere, their mass spectrometry analysis, and the subsequent effect on plant growth. AHLs identified in SAL-17 and SAL-21 strains include 3-OH-C5-HSL, C6-HSL, 3-OH-C6-HSL, 3-oxo-C7-HSL, C10-HSL, 3-oxo-C10-HSL, 3-oxo-C10-HSL + H_2_O, 3-OH-C10-HSL, and 3-oxo-C12-HSL + H_2_O. There are various AHLs reported from genus *Aeromonas* (Supplementary Table S1), but six AHLs (3-OH-C5-HSL, 3-OH-C6-HSL, 3-oxo-C7-HSL, 3-oxo-C10-HSL, 3-OH-C10-HSL, and 3-oxo-C12-HSL) identified in this study (Table 3) were not reported earlier, a feature that makes SAL-17 and SAL-21 different from other *Aeromonas* species. Being different in the genus *Aeromonas*, we deduced the function of these six AHLs from already published studies where they have been detected from other bacterial species. The functional annotation of the detected AHLs (Table 4) in the *Aeromonas* spp. SAL-17 and SAL-21 showed that these AHLs are mainly involved in the induction of systemic resistance against various pathogens, synthesis of phytohormones, and plant growth promotion. Exclusively C6-HSL plays its role in the induction of systemic resistance against biotic and abiotic stresses, root colonization, and biofilm formation by bacteria, root growth, and development. C6-HSL, 3-oxo-C-10-HSL, and 3-oxo-C12-HSL have a combined well-defined role against salt stress *via* the enhanced activity of superoxide dismutase (SOD), peroxidase (POD), and catalase (CAT) enzymes. 3-OH-C6-HSL is known to induce the production of antifungal metabolites by root-colonizing microbes, whereas 3-oxo-C7-HSL assists in the colonization process, and it may serve as biocontrol component in the rhizosphere. The signal molecules 3-OH-C6-HSL, 3-oxo-C7-HSL, 3-oxo-C-10-HSL, and 3-oxo-C12-HSL have been shown to protect against biotic and abiotic stresses. Furthermore, their role in biofilm formation, root colonization, and development of lateral and primary roots has also been described. The signal molecules C10-HSL and 3-OH-C1-HSL have been reported to control the primary and the secondary growth of root and colonization and the induction of plant defense response. Furthermore, they have a well-defined role in root growth and development, along with a significant impact on photosynthesis, induced stress resistance, and plant hormone signaling pathways. 3-Oxy-C10-HSL helps in biofilm formation and improves the growth of adventitious roots and the expression of indole-3-acetic acid (IAA)-responsive genes, while 3-OH-C10-HSL mediates plant root colonization, growth promotion, and induction of defense response. 3-Oxo-C12-HSL serves in biofilm formation and expression of stress-related, hormonal, and regulatory genes. The function of 3-OH-C5-HSL is not mentioned in the literature, but studies suggest that it might have some role in symbiosis as stated by Zarkani et al. (2013). The detection of this AHL type from *Aeromonas* spp. in the present study suggests that it might have some other functions in plants rather than just symbiosis. The same AHL from different bacteria exhibits fairly similar functions; detailed molecular studies are required for the validation of these functions.

The root colonization potential of a bacterium is necessary to develop a successful interaction with plant, and bacteria use AHL-mediated synchronized response to design and establish an efficient interaction between the host and its associated symbionts (Schenk and Schikora, 2015). Both strains (SAL-21 and SAL-17) possess IAA production ability (Rajput et al., 2018) and AHL production (this study) and exhibited their colonization ability on wheat roots in different experiments, i.e., confocal microscope analysis and plate assay. The confocal analysis showed that bacterial colonization is a little affected in the presence of salt. Furthermore, a modified plate overlay assay validated the root colonization and purple color, along with the growing seedling root, displaying that AHLs are being produced and might have a robust role in root development. AHL-mediated root colonizing ability has been previously reported in rhizobia and genus *Pantoea* (Sutherland, 2001; Koutsoudis et al., 2006).

The root overlay assay revealed a likely role of AHLs in early root growth, which was further confirmed in a plate assay where purified AHLs were applied in growth medium and seeds were grown without bacterial inoculation. The data regarding root morphology establish the fact that the increase in root growth is the function of AHLs rather than of IAA. The plate overlay assay of extracted AHLs, RP-TLC analysis, and SPE steps ruled out the likelihood of the presence of IAA traces in the AHL extracts. The AHL extracts of these strains contain C6-HSL, which has a well-reported function in primary root elongation, auxin/cytokinin ratio alteration, transcriptional regulation, and biomass improvement (von Rad et al., 2008). The other AHL in the extract was C10-HSL, which enhances lateral root growth (Bai et al., 2012; Zhao et al., 2015) and shoot growth (Götz et al., 2007) in different plants. Purified AHL extracts from *Bradyrhizobium* sp. strain SR-6, which produces a wide variety of AHLs including C6-HSL, C10-HSL, 3-oxo-C10-HSL, 3-oxo-C12-HSL, *etc*., significantly improved root hair development in wheat, along with increased nodulation in soybean (Ali et al., 2016).

Plant response was further evaluated by strain inoculation in saline soil. An IAA-positive but AHL-non-producing *Aeromonas* sp. strain SAL-12 indigenous to saline soil (Rajput et al., 2018) was used for comparison of inoculation response. SAL-12, along with both AHL-producing *Aeromonas* spp. SAL-17 and SAL-21, also exhibits ACC deaminase activity. IAA is a plant hormone that is involved in the stimulation of plant growth, and ACC deaminase has a well-known function in salt stress mitigation *via* the cutting synthesis of ethylene (Ahmad et al., 2011; Arshadullah et al., 2017; Sarkar et al., 2018; Afridi et al., 2019; Bharti and Barnawal, 2019; del Carmen Orozco-Mosqueda et al., 2020). Both these traits are a characteristic feature of any PGP candidate species because some studies have shown a synergistic effect of bacterial IAA and ACC deaminase. Both IAA and ACC deaminase have direct positive effects on root growth and root hair development, which help to enhance water and nutrient absorption from the soil (Príncipe et al., 2007). Our plant inoculation data demonstrated the significance of AHLs for plant growth under stress along with the role of IAA and ACC deaminase. For instance, if SAL-17 produces a wide variety of AHLs and higher IAA than SAL-21 and SAL-12, the response toward SAL-17 single inoculation and a mix inoculation (SAL-17 + SAL-21) was more pronounced on different biochemical, physiological, and growth parameters of wheat. AHLs directly or indirectly induce stress resistance in plants through QS-mediated production of metabolites (Ryu et al., 2013). It has been reported that inoculation of 3-oxo-C12-HSL-producing bacterial strains induce salt stress tolerance, metabolic regulation, and phytohormone response in tomato and *Medicago truncatula* (Mathesius et al., 2003; Barriuso et al., 2008). The data from this study have presented many folds increase in the NR activity. NR is the enzyme responsible for nitrate assimilation and the production of NO in plants (Chamizo-Ampudia et al., 2017). NR-mediated NO also has been reported as a key signaling molecule in leaf shape development (Pan Q.-N. et al., 2019), root geotropism (Vazquez et al., 2019), and various stress responses by plants. Although purified AHLs were not used in the experiment, still we speculate that AHLs have some role in the regulation of NR activity, which in turn induces salt stress tolerance in wheat because NR activity and proline contents directly correlate with AHL production. PGPR has a documented role to accumulate higher proline in plants under stress (Jha and Subramanian, 2014; Kumari et al., 2015; Singh and Jha, 2016b), and this study advocates this role along with some plausible role of AHLs, although it is still unclear how plants perceive these signals and how many are responsible to elicit these responses in plants. However, plants inoculated with the bacterial consortium (SAL-17 + SAL-21) showed a significantly (*P* ≤ 0.05) higher response and a maximum percent increase for all parameters in both genotypes of wheat. Further experiments using purified and inclusive inoculum for each kind of AHL with different concentrations and gene knockout studies can elucidate the role of individual AHLs on plant growth.

The data regarding stomatal conductance, transpiration rate, and photosynthetic pigments show that these parameters were significantly increased (*P* ≤ 0.05) in inoculated plants in both wheat genotypes under salt stress, wherein plants inoculated with AHL-producing *Aeromonas* sp. strains (SAL-17 and SAL-21) showed a better response in terms of percent increase than the plants inoculated with non-AHL-producing *Aeromonas* sp. strain (SAL-12). This may be attributed to the contribution of AHLs in the overall plant response. Salinity usually causes osmotic stress in plants (Upadhyaya et al., 2013), which leads to stomatal closure by altering the turgor potential of the guard cells. It is a feedback process to prevent water loss *via* transpiration (Buckley and Mott, 2013), but it also blocks the passage for CO_2_, causing the photosynthetic activity to decrease. Not by themselves but the degraded products of AHLs have been reported to enhance stomatal conductance and transpiration rate in mung bean (Joseph and Phillips, 2003) because AHLs are not stable in the soil and readily degrade into their active constituents (Wang and Leadbetter, 2005). As acyl-HSLs in the rhizosphere are degraded, the bioavailability of nutrients to the roots and root-associated bacteria increases, which indirectly increases transpiration and growth (Joseph and Phillips, 2003). PGPR-mediated improvement in chlorophyll pigments and overall photosynthetic capacity is well established (Babaei et al., 2017; Afridi et al., 2019; Azarmi-Atajan and Sayyari-Zohan, 2020; Ji et al., 2020). A meta-analysis of 561 studies has suggested the positive role of PGPR inoculation in K^+^/Na^+^ ratio ion homeostasis, Na^+^ exclusion, and enhanced photosynthetic activity (Pan J. et al., 2019). Moreover, inoculated *Aeromonas* spp. strains also produce IAA.

Along with the biochemical and the physiological parameters, the growth and the yield parameters of plants were significantly (*P* ≤ 0.05) improved in inoculated plants of both genotypes as compared to the non-inoculated control. It could be an accumulative effect of multiple PGP and stress tolerance traits and a wide range of AHLs. A significant contribution of AHLs becomes obvious when results for different growth parameters and yield from plants inoculated with AHL-producing strains SAL-21 and SAL-17 were compared with those of non-AHL-producing strain SAL-12. The role of PGPR in stress tolerance amelioration, plant growth, and yield improvement of several crops is well established (Van Loon, 2007). Plant-beneficial bacteria play a key role in the improvement of crop growth, nutrition, and yields and in sustaining soil productivity with low input of chemical fertilizers under stress (Islam et al., 2016; Yasmeen et al., 2019).

The results of the current study advocate that plant-associated beneficial *Aeromonas* spp. strains have a significant role in salt stress mitigation and overall plant growth improvement. Moreover, stress-resilient PGPR is the best choice to be used as inoculants under stressful conditions because they can sustain stress and maintain their PGP traits as well. This study indicates the contribution of AHLs in stress tolerance induction, but other plant-growth-promoting factors cannot be ruled out completely. Furthermore, plants can be engineered for AHL production to foster their interaction with beneficial microbes as previously reported for bioengineered plants (Scott et al., 2006). A study in which tomato plants were inoculated with AHL-producing strains and also bioengineered for production of short-chain and long-chain AHLs has concluded that AHLs promote plant growth and confer protection against salt stress (Barriuso et al., 2008). The current study opens future directions for the researchers to study the AHL regulation of microbial process and plant response modulation through induction of stress-responsive genes and signaling pathways.

## Conclusion

This study has demonstrated a wide range of AHL production by the halotolerant plant-growth-promoting *Aeromonas* spp. strains SAL-17 and SAL-21, in which six are unique to the two strains being reported. Exogenous application of purified AHLs significantly increased the root morphology in wheat. Both strains showed the potential to colonize wheat roots and stimulate substantial growth under saline conditions in two different wheat genotypes. The inoculated plants showed higher proline contents, transpiration rate, stomatal conductance, chlorophyll contents, and nitrate reductase activity. The overall growth stimulation may be attributed to a synergistic response of the IAA, ACC-deaminase activity, and AHL production, of which the role of AHLs seems imperative. Future research involving AHL-deficient mutants, use of synthetic AHLs, and AHL-engineered plants will further validate the role of AHLs because, in this study, the comparison of inoculation results for the AHL-producing strains with a strain missing AHL production suggested their significant contribution toward salt stress mitigation and plant growth improvement. A comparison with phenotypically AHL-negative strain derivative using lactonase constructs would result in more direct evidence because the genetic background would be the same. This study concludes that multi-trait, non-pathogenic *Aeromonas* spp. strains are candidates of choice for the production of inoculum for saline soils. The strains should be used/released in the field only after implementation of biosafety parameters because some aeromonads have clinical significance. The study is of prime importance because 45 million hectares of salt-affected soil direly need an efficient solution for better cropping on a larger scale.

## Data Availability Statement

The data can be found at NCBI [SAL-17 (Accession No. HG763857) and SAL-21 (Accession No. HG763858)].

## Author Contributions

MN analyzed the data and wrote the manuscript. AA performed the AHL experiments as part of her MPhil research work. LR conducted the strain characterization and the pot experiments as part of her Ph.D. research. KF performed the confocal studies. SU helped in the AHL screening experiment. MA helped in the pot experiments. AI conceived and supervised the whole study and edited the manuscript. All the authors read and approved the final version of the manuscript.

## Conflict of Interest

The authors declare that the research was conducted in the absence of any commercial or financial relationships that could be construed as a potential conflict of interest.

## Supplementary Material

Supplementary Figure 1 Phylogenetic relationship of *Aeromonas* spp. Sal-12, Sal-17, and Sal-21 based on the sequences of 16S rRna along with closely related sequences obtained from GenBank. Boot strap value was 1,000, and nucleotide sequence divergence is shown by a bar. The tree was generated in Mega6 using maximum likelihood method.

Supplementary Figure 2 Grain yield response to proline contents and nitrate reductase activity as a function of bacterial inoculation in two different wheat genotypes grown in both saline and normal soil. Data from all treatments were jointly loaded on the graph to evaluate the overall response. The graph shows the quadratic relationship of grain yield to plant proline and nitrate reductase activity, with significantly high *R*^2^ values.

Supplementary Table 1 Occurrence and functional annotation of acyl homoserine lactones in spent culture supernatant of *Aeromonas* spp. reported in literature.
