# Supplementary material for: Growth-Stimulatory Effect of Quorum Sensing Signal Molecule N-Acyl-Homoserine Lactone-Producing Multi-Trait Aeromonas spp. on Wheat Genotypes Under Salt Stress
Source: Front Microbiol. 2020 Sep 29;11:553621. doi: 10.3389/fmicb.2020.553621 (PMC7550764; doi:10.3389/fmicb.2020.553621)
Supplement: Supplementary Figure 1 — Phylogenetic relationship of Aeromonas spp. Sal-12, Sal-17, and Sal-21 based on the sequences of 16S rRna along with closely related sequences obtained from GenBank. Boot strap value was 1,000, and nucleotide sequence divergence is shown by a bar. The tree was generated in Mega6 using maximum likelihood method. [file Data_Sheet_1.PDF]

Growth stimulatory effect of quorum sensing signal molecule N-Acyl-homoserine lactones-producing multi-trait *Aeromonas* spp. on wheat genotypes under salt-stress

Muhammad Shoib Nawaz[1], Ayesha[1], Lubna Rajput[1, 2], Kaneez Fatima[1, 3], Sami Ullah[1, 4], Muhammad Ahmad, Asma Imran[1*]

[1]Institute for Biotechnology and Genetic Engineering (NIBGE), P.O. Box 577, Jhang Road, Faisalabad, Pakistan
[2]Plant Physiology Section, Agriculture Research Institute, Tandojam, Pakistan
[3]University of Management and Technology (UMT), C-II Block, Phase 1, Johar Town, Lahore, Pakistan
[4]University of Bagh, Azad Jammu, and Kashmir, Pakistan

*** Correspondence:**
Asma Imran; asmaaslam2001@yahoo.com

Table S1 Occurrence and functional annotation of AHLs in spent culture supernatant of *Aeromonas* spp. in literature

Strains	Isolates origin	AHLs detection and quantification methods	AHLs produced	Findings of study	References
A. hydrophila AH-1	Natural isolate	TLC (CV026)/HPLC-HR-MS	C4-HSL++, C6-HSL	Increased serine protease activity (virulence determinants)	(Swift et al. 1997)
A. salmonicida NCIMB 1102[T]	Salmon isolate				
A. hydrophila AH-1	Natural isolate	TLC (CV026, pSB536)/HPLC-HR-MS-NMR	C4-HSL++, C6-HSL	Increased exoprotease production (biofilm determinants)	(Lynch et al. 2002)
Aeromonas sp. (n = 3)	Activated sludge isolate	TLC (CV026, pZLR4)	C6-HSL, or 3-oxo-C6-HSL, C4-HSL, C6-HSL, 3-oxo-C8-HSL or C8-HSL	Putative role in bioaggregate formation	(Morgan-Sagastume et al. 2005)
A. hydrophila ATCC7966[T]	Diseased fish isolate	TLC (CV026, pZLR4)/HPLC-HR-MS	C4-HSL	Regulate expression of virulence phenotypes	(Bruhn et al. 2005)
A. hydrophila 96-3-35			C4-HSL, C6-HSL		
A. salmonicida NCIMB 1110			C4-HSL, C6-HSL		
A. salmonicida 02-9-1			C4-HSL, C10-HSL, C6-HSL, 3-oxo-C6-HSL		
A. hydrophila strains (n = 8)	Faeces and foods isolate	TLC (CV026)	C4-HSL	*Aeromonas* strains produced C4-HSL under a different salt, temperature and pH conditions.	(Medina-Martinez et al. 2006)
A. caviae strains (n = 5)	Foods isolate				
A. hydrophila	Natural isolate	GC-MS	C8-HSL, C12-HSL, C14-HSL	GC-MS is more comprehensive and reliable technique for AHLs detection	(Cataldi et al. 2007)
A. salmonicida	Salmon isolate		C8-HSL, C10-HSL C12-HSL, C14-HSL		
A. culicicola 3249[T]	Mosquito midgut isolate	GC-MS	C6-HSL, isoC7-HSL, 3OH-isoC7-HSL, 3OH-C8-HSL, 3OH-isoC9-HS	First report of methyl-branched AHLs in bacterial QS systems and power of GC-MS technique to identify unknown AHLs	(Thiel et al. 2009)
A. hydrophila (n = 4)	Peritoneal fluid, pus, stool and blood	TLC (CV026)	C4-HSL	C4-HSL is abundantly produces as compared to C6-HSL in clinical isolates	(Chan et al. 2011)
A. hydrophila (n = 6)			C4-HSL, C6-HSL		
A. hydrophila (n = 10)			C4-HSL, putative C5-HSL, C6-HSL		
A. sobria 159	Stool and bile		C4-HSL		
A. sobria 222			C6-HSL, 2 UI-HSL		

A. salmonicida subsp. *Achromogenes* Keldur265-87	Diseased fish isolate	TLC (CV026)/HPLC-HR-MS	C4-HSL	Virulence and pigment production are regulated by C4-HSL produced.	(Schwenteit et al. 2011)
Aeromonas sp. strain GC1	Activated sludge isolate	TLC (CV026)/NSI-MS/LC-ESI-MS	C4-HSL, C6-HSL, C8-HSL, C12-HSL	AHLs dependent production of extracellular degradative enzymes such as chitinase, elastase lipase, and cellulase is reported.	(Chong et al. 2012)
A. tjernbergiae B017	Urban river biofilm isolates	TLC (CV026, A136)	C6-HSL	*Aeromonas* sp. comprise a major AHLs producing group of river biofilm.	(Huang et al. 2012)
A. aquariorum B2M05			C4-HSL, 1 UI-HSL		
A. hydrophila B015			C4-HSL, C6-HSL, 1 UI-HSL		
A. hydrophila B1M18			C4-HSL, 2 UI-HSL		
A. jandaei B087			C4-HSL, 1 UI-HSL		
A. media Bill			C4-HSL, 2 UI-HSL		
A. mediaB026					
A. media B1M53			C4- HSL, C8-HSL, 3-OH-C8-HSL		
A. salmonicida B079			C4-HSL, 1 UI-HSL		
A. veronii B025			3-OXO-C8-HSL, 1 UI-HSL		
A. veronii MTCC 3249, originally isolated as *A. culicicola*	Mosquito midgut isolate	TLC (CV026)/HPLC-MS/NMR/IR	C4-HSL++, C6-HSL, C14-HSL, 6-carboxy-C6-HSL	Single strain produces so many AHLs under control of single system and first report of carboxyl-AHL production in *Aeromonas* genus and or any bacterial genera.	(Jangid et al. 2012)
A. hydrophila strains (n = 35)	Activated sludge isolate	TLC (CV026, VIR07)	C4-HSL, C6-HSL	*Aeromonas* spp. comprise a major AHLs producing group of activated sludge possibly affecting waste water treatment process.	(Ochiai et al. 2013)
A. punctate strains (n = 16)					
A. sobria strains (n = 22)					
A. media strains (n = 24)					
A. veronii strains (n = 4)					
A. jandaei strains (n = 1)					
A. hydrophila strains (n = 24)	fresh water or diseased fish	TLC (CV026)	C4-HSL, C6-HSL	83 % of the isolates produced ß haemolysin and	(Chu et al. 2013)

				produced cytotoxic activity, 75% shown proteolytic activity and 50% displayed DNase activity.	
A. hydrophila S1-073	Rhizosphere of *Salix babylonica* (willow)	TLC (CV026, A136)	C4-HSL, C6-HSL, 1 UI-HSL	*Aeromonas* represented the most abundant group (66.7 %) of willow rhizosphere	(Zeng et al. 2014)
A. media S1_063			C4-HSL, 2 UI-HSL		
A. media S1_008			Not determined		
A. media S1_060					
A. veronii S1_078					
A. veronii S1_080					
A. trota S2_007	Rhizosphere of *Phragmites australis* (reed)		C4-HSL, 1 UI-HSL	*Aeromonas* represented 22.2% of reed rhizosphere	
A. aquariorum S2_004			Not determined		
A. Caviae Strain YL12	Compost isolate	TLC (CV026)/MALDI-TOF-MS	C4-HSL, C6-HSL	AHLs production was density dependent.	(Lim et al. 2014)
Aeromonas spp. (n = 22)	Different foods isolates	TLC (CV026, JB523, NT1)	C4- HSL, putative C5-HSL, C6-HSL, C7-HSL	All the food isolates of *Aeromonas* shown diverse profile of AHLs and first-time reporting C7-HSL. No co-relation was found between AHLs profile and biofilm formation.	(Nagar et al. 2015)
A. hydrophila strain KOR1	Mangrove rhizosphere soil isolate	Whole-genome shotgun project	C4-HSL, C6-HSL	Putative role of facilitator in nitrogen fixation	(Yin et al. 2015)
A. hydrophila KCTC 11533	Surface water	TLC (CV026)	C4-HSL	Salinity affects both AHLs production and biofilm formation.	(Jahid et al. 2015)
Aeromonas spp. (n = 5)	Spoiled refrigerated turbot	RP-TLC (CV026)/GC-MS	3-oxo-C6-HSL, C8-HSL, C10-HSL, C12-HSL	AHLs production was density dependent and exogenous addition of AHLs reduced biofilm formation.	(Zhang et al. 2016)
A. sobria AS7	Spoiled turbot	TLC (CV026, A136)/GC-MS	C4-HSL, C6-HSL, C8-HSL, C10-HSL, C12-HSL	C4-HSL and C8-HSL were found to be involved in population growth and while C6-HSL was not found to be involved in spoilage.	(Li et al. 2016)

Aeromonas sp. strain GLY-2107	Lake Taihu isolate	TLC (CV026)/UPLC-HRMS/MS	C4-HSL, C6-HSL	AHLs regulate the algicidal activity.	(Guo et al. 2016)
A. veronii bv. *veroni*	Fermented surimi	LC–MS/MS	C4-HSL, C6-HSL, C8-HSL	AHL-based quorum sensing system of *Aeromonas* spp. involved in the regulation of spoilage.	(Zhao et al. 2018)
A. veronii LP-11	Sturgeon fish isolate	TLC (CV026 & KYC55)/ HPLC/QqQ-MS/ QTOF-MS	C6-HSL, 3-oxo-C8-HSL, 3-OH-C8-HSL, C8-HSL	Putative role AHLs in sturgeon spoilage	(Gui et al. 2018)

AHL, N-acyl homo serine lactone; HSL, homo serine lactone; UI-HSL, Unidentified homo serine lactone; HPLC-HR-MS, high performance liquid chromatography/high resolution mass spectrometry; GC-MS, Gas chromatography-mass spectrometry; HPLC-MS-NMR, high performance liquid chromatography/mass spectrometry-nuclear magnetic resonance; NSI-MS, nano spray ionization-mass spectrometry; LC-ESI-MS, liquid chromatography/electro spray ionization-mass spectrometry; TLC, thin layer chromatography; RP-TLC, reverse plate-thin layer chromatography; CV026, reporter strain *Chromobacterium violaceum* CV026; pZLR4, reporter strain *Agrobacterium tumefaciens* NTL4 (pzLR4); A136, reporter strain *Agrobacterium tumefaciens* A136; KYC55, reporter strain *Agrobacterium tumefaciens* KYC55; ++, major AHLs produced.

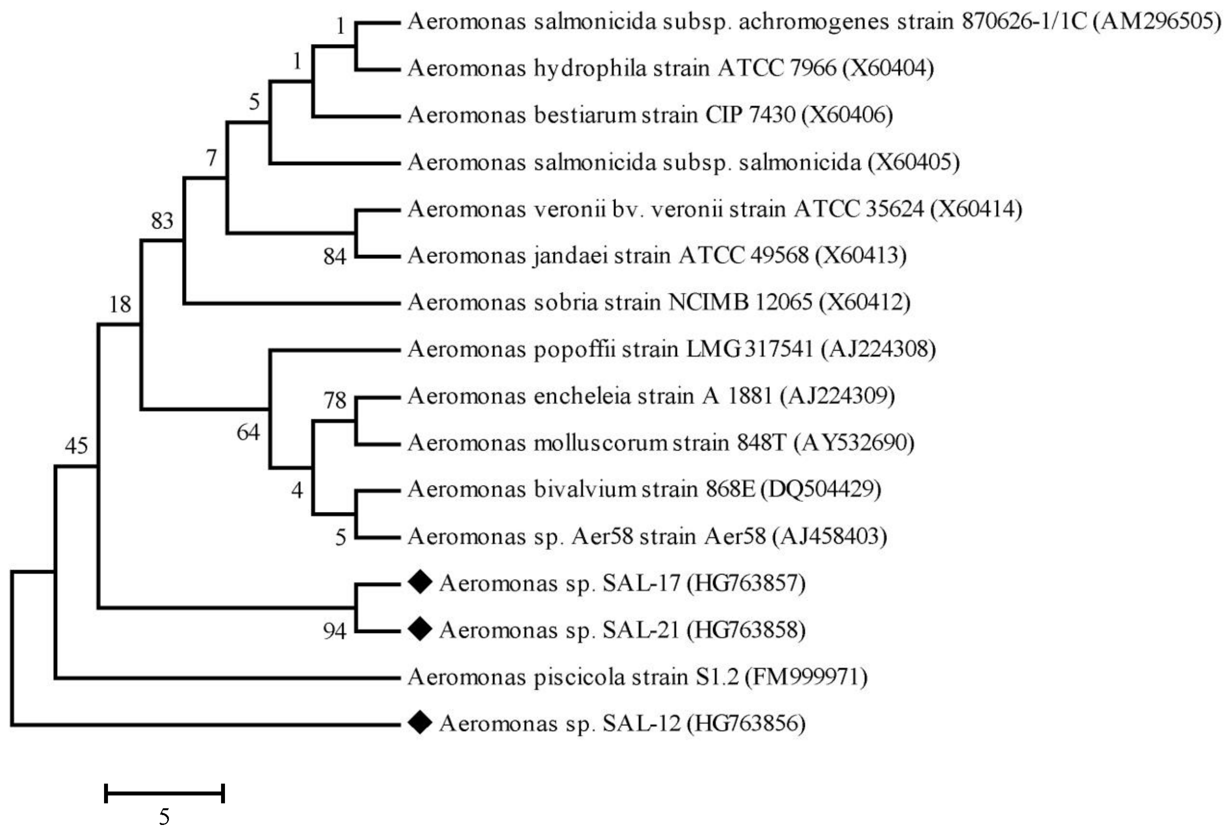

Figure S1: Phylogenetic relationship of *Aeromonas* spp. SAL-12, SAL-17 and SAL-21 based on the sequences of 16S *rRNA* along with closely related sequences obtained from GenBank. Boot strap value was 1000 and nucleotide sequence divergence is shown by bar. The tree was generated in MEGA6 using maximum likelihood method.

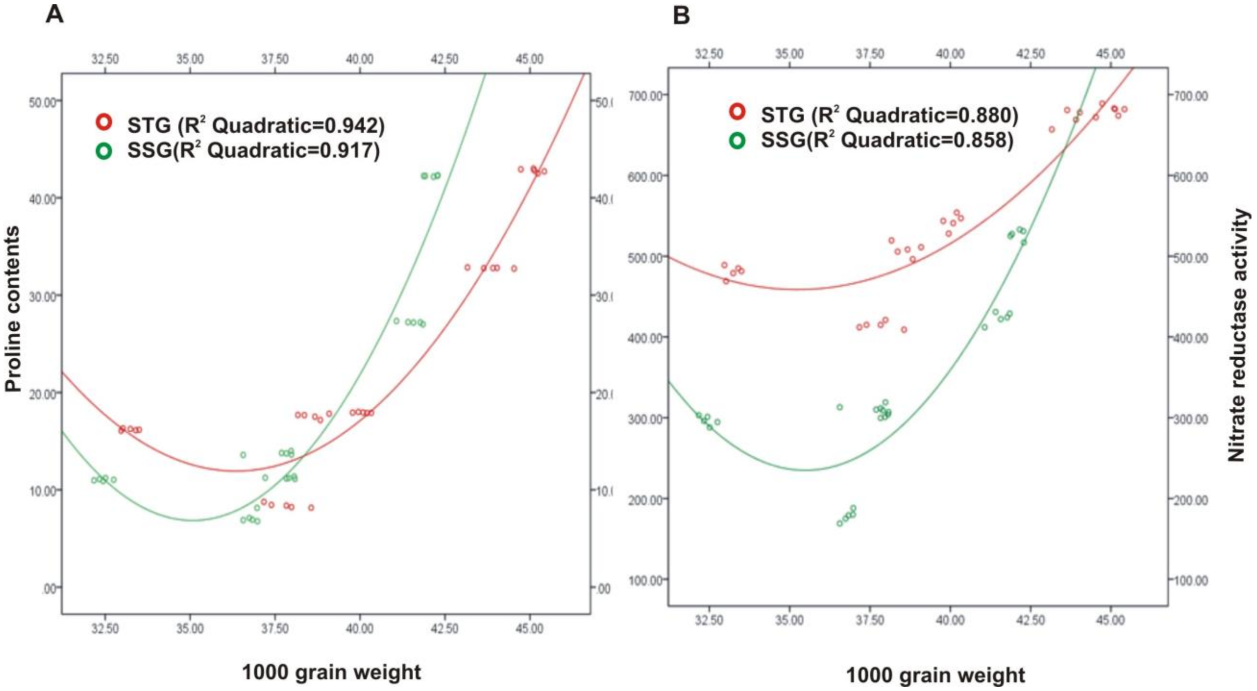

Figure S2: Grain yield response to proline contents and nitrate reductase activity as a function of bacterial inoculation in two different wheat genotypes grown in two saline and normal soil. The data from all treatments was jointly loaded on the graph to evaluate the overall response. Graph shows the quadratic relationship of grain yield to plant proline and nitrate reductase activity with significant high R^2-values.

Literature cited:

Bruhn, J.B., Dalsgaard, I., Nielsen, K.F., Buchholtz, C., Larsen, J.L., Gram, L. (2005). Quorum sensing signal molecules (acylated homoserine lactones) in gram-negative fish pathogenic bacteria. *Diseases of Aquatic Organisms* 65(1), 43-52.

Cataldi, T.R., Bianco, G., Palazzo, L., Quaranta, V. (2007). Occurrence of N-acyl-L-homoserine lactones in extracts of some Gram-negative bacteria evaluated by gas chromatography–mass spectrometry. *Analytical Biochemistry* 361(2), 226-235.

Chan, K-G,, Puthuchearym S,D,, Chanm X-Y., Yin, W-F., Wong, C-S., Too, W-S.S., Chua, K-H. (2011). Quorum sensing in *Aeromonas* species isolated from patients in Malaysia. *Current Microbiology* 62(1), 167-172.

Chong, G., Kimyon, O., Rice, S.A., Kjelleberg, S., Manefield, M. (2012). The presence and role of bacterial quorum sensing in activated sludge. *Microbial Biotechnology* 5(5), 621-633.

Chu, W., Liu, Y., Jiang, Y., Zhu, W., Zhuang, X. (2013). Production of N-acyl homoserine lactones and virulence factors of waterborne *Aeromonas hydrophila*. Indian Journal of Microbiology 53(3), 264-268.

Gui, M., Liu, L., Wu, R., Hu, J., Wang, S., Li, P. (2018). Detection of new quorum sensing N-acyl homoserine lactones from *Aeromonas veronii*. *Frontiers in Microbiology* 9, 1712.

Guo, X., Liu, X., Wu, L., Pan, J., Yang, H. (2016). The algicidal activity of *Aeromonas* sp. strain GLY-2107 against bloom-forming *Microcystis aeruginosa* is regulated by N-acyl homoserine lactone-mediated quorum sensing. *Environmental Microbiology* 18(11), 3867-3883.

Huang, Y., Zhang, J., Yu, Z., Zeng, Y., Chen, Y. (2012). Isolation and characterization of acyl homoserine lactone–producing bacteria during an urban river biofilm formation. Archives of *Microbiology* 194(12), 1043-1048.

Jahid, I.K., Mizan, M.F.R., Ha, A.J., Ha, S-D. (2015). Effect of salinity and incubation time of planktonic cells on biofilm formation, motility, exoprotease production, and quorum sensing of *Aeromonas hydrophila*. *Food Microbiology* 49, 142-151.

Jangid, K., Parameswaran, P.S., Shouche, Y.S. (2012). A variant quorum sensing system in *Aeromonas veronii* MTCC 3249. *Sensors* 12(4), 3814-3830.

Li, T., Cui, F., Bai, F., Zhao, G., Li, J. (2016). Involvement of acylated homoserine lactones (AHLs) of *Aeromonas sobria* in spoilage of refrigerated turbot (*Scophthalmus maximus* L.). *Sensors* 16(7), 1083.

Lim, Y-L., Ee, R., Yin, W-F., Chan, K-G. (2014). Quorum sensing activity of *Aeromonas caviae* strain YL12, a bacterium isolated from compost. *Sensors* 14(4), 7026-7040.

Lynch, M.J., Swift, S., Kirke, D.F., Keevil, C.W., Dodd, C.E., Williams, P. (2002). The regulation of biofilm development by quorum sensing in *Aeromonas hydrophila*. *Environmental Microbiology* 4(1), 18-28.

Medina-Martinez, M., Uyttendaele, M., Demolder, V., Debevere, J. (2006). Influence of food system conditions on N-acyl-L-homoserine lactones production by *Aeromonas* spp. International *Journal of Food Microbiology* 112(3), 244-252.

Morgan-Sagastume, F., Boon, N., Dobbelaere, S., Defoirdt, T., Verstraete, W. (2005). Production of acylated homoserine lactones by *Aeromonas* and *Pseudomonas* strains isolated from municipal activated sludge. *Canadian Journal of Microbiology* 51(11), 924-933.

Nagar, V., Sinha, V., Bandekar, J.R. (2015). Diverse Profiles of N-acyl Homoserine l-Lactones, Biofilm, Virulence Genes and Integrons in Food-Borne *Aeromonas* Isolates. *Journal of Food Science* 80(8), 1861-1870.

Ochiai, S., Morohoshi, T., Kurabeishi, A., Shinozaki, M., Fujita, H., Sawada, I., Ikeda, T. (2013). Production and degradation of N-acylhomoserine lactone quorum sensing signal molecules in bacteria isolated from activated sludge. *Bioscience, Biotechnology, and Biochemistry* 77(12), 2436-2440.

Rajput, L., Imran, A., Mubeen, F., Hafeez, F.Y. (2018). Wheat (*Triticum aestivum* L.) growth promotion by halo-tolerant PGPR-consortium. *Soil and Environment* 37(2), 178-189.

Schwenteit, J., Gram, L., Nielsen, K.F., Fridjonsson, O.H., Bornscheuer, U.T., Givskov, M., Gudmundsdottir, B.K. (2011). Quorum sensing in Aeromonas salmonicida subsp. achromogenes and the effect of the autoinducer synthase AsaI on bacterial virulence. *Veterinary Microbiology* 147(3-4), 389-397.

Swift, S., Karlyshev, A.V., Fish, L., Durant, E.L., Winson, M.K., Chhabra, S.R., Williams, P., Macintyre, S., Stewart, G. (1997). Quorum sensing in *Aeromonas hydrophila* and *Aeromonas salmonicida*: identification of the LuxRI homologs AhyRI and AsaRI and their cognate N-acylhomoserine lactone signal molecules. *Journal of Bacteriology* 179(17), 5271-5281.

Thiel, V., Kunze, B., Verma, P., Wagner-Döbler, I., Schulz, S. (2009). New structural variants of homoserine lactones in bacteria. *Chem BioChem* 10(11), 1861-1868.

Yin, M., Ma, Z., Cai, Z., Lin, G., Zhou, J. (2015). Genome sequence analysis reveals evidence of quorum-sensing genes present in *Aeromonas hydrophila* strain KOR1, isolated from a mangrove plant (Kandelia obovata). *Genome Announc* 3(6), e01461-15.

Zeng, Y., Yu, Z., Huang, Y. (2014). Combination of culture-dependent and-independent methods reveals diverse acyl homoserine lactone-producers from rhizosphere of wetland plants. *Current Microbiology* 68(5), 587-593/

Zhang, C., Zhu, S., Jatt, A-N., Zeng, M. (2016). Characterization of N-acyl homoserine lactones (AHLs) producing bacteria isolated from vacuum-packaged refrigerated turbot (*Scophthalmus maximus*) and possible influence of exogenous AHLs on bacterial phenotype. *The Journal of General and Applied Microbiology* 62(2), 60-67.

Zhao, D., Lyu, F., Liu, S., Zhang, J., Ding, Y., Chen, W., Zhou, X. (2018). Involvement of bacterial quorum sensing signals in spoilage potential of *Aeromonas veronii* bv. veronii isolated from fermented surimi. *Journal of Food Biochemistry* 42(2), e12487 .
